# Mitochondrial dysfunction associated with TANGO2 deficiency

**DOI:** 10.1038/s41598-022-07076-9

**Published:** 2022-02-23

**Authors:** Paige Heiman, Al-Walid Mohsen, Anuradha Karunanidhi, Claudette St Croix, Simon Watkins, Erik Koppes, Richard Haas, Jerry Vockley, Lina Ghaloul-Gonzalez

**Affiliations:** 1 Division of Genetic and Genomic Medicine, Department of Pediatrics, University of Pittsburgh, Pittsburgh, PA USA; 2 Department of Human Genetics, Graduate School of Public Health, University of Pittsburgh, Pittsburgh, PA USA; 3 Department of Cell Biology, Center for Biologic Imaging, University of Pittsburgh, Pittsburgh, PA USA; 4 Division of Pediatric Neurology, Departments of Neurosciences and Pediatrics, University of California San Diego and Rady Children’s Hospital-San Diego, San Diego, CA USA

**Keywords:** Genetics, Medical research, Diseases

## Abstract

Transport and Golgi Organization protein 2 Homolog (*TANGO2*)-related disease is an autosomal recessive disorder caused by mutations in the *TANGO2* gene. Symptoms typically manifest in early childhood and include developmental delay, stress-induced episodic rhabdomyolysis, and cardiac arrhythmias, along with severe metabolic crises including hypoglycemia, lactic acidosis, and hyperammonemia. Severity varies among and within families. Previous studies have reported contradictory evidence of mitochondrial dysfunction. Since the clinical symptoms and metabolic abnormalities are suggestive of a broad dysfunction of mitochondrial energy metabolism, we undertook a broad examination of mitochondrial bioenergetics in TANGO2 deficient patients utilizing skin fibroblasts derived from three patients exhibiting *TANGO2*-related disease. Functional studies revealed that TANGO2 protein was present in mitochondrial extracts of control cells but not patient cells. Superoxide production was increased in patient cells, while oxygen consumption rate, particularly under stress, along with relative ATP levels and β-oxidation of oleate were reduced. Our findings suggest that mitochondrial function should be assessed and monitored in all patients with TANGO2 mutation as targeted treatment of the energy dysfunction could improve outcome in this condition.

## Introduction

*TANGO2*-related disease (MIM#616878) is an autosomal recessive disorder caused by mutations in the *TANGO2* (transport and Golgi organization protein 2 homolog) gene, also known as *C22orf25* (chromosome 22q11.21). It is characterized by episodic rhabdomyolysis, usually triggered by stress such as illness or fasting, global developmental delay, encephalopathy, cardiac arrhythmias including long QT Syndrome, and severe metabolic crises including hypoglycemia, lactic acidemia, elevated CPK, and hyperammonemia. Hypothyroidism has also been identified in numerous patients with the disorder^1,2^. Variable blood acylcarnitine profiles have been reported in patients, normal in some but abnormal in others with elevated C3, C10, C12, C14:1, C14:2, C16, C18-carnitine species and some acylcarnitine hydroxy derivatives^3,4^. Allelic heterogeneity both in the presence and severity of symptoms along with intrafamilial variability have been reported, suggesting the presence of modulating genes or variable tissue damage induced by patient specific crises^1,5^.

Multiple mutations in the *TANGO2* gene have been reported in affected patients. In patients of European descent, the most common mutation is a deletion of exons 3–9, often homozygous, resulting in a truncated, non-functional protein^1,3,4^. The most common genotype in patients of Hispanic/Latino descent is a homozygous c.460G > A (p.Gly154Arg) missense variant in exon 7, while three consanguineous Arab patients, including two siblings, have a homozygous deletion of exons 4–6^1,3^. In addition, numerous other variants have been reported including a splice site variant in intron 7, c.605 + 1G > A^3,5^.

While the specific function of the TANGO2 protein is still unknown, RNA-mediated interference screens in *Drosophila* S2 cells showed that the depletion of TANGO2 protein caused Golgi membranes to fuse with the endoplasmic reticulum (ER)^6^. This finding led to the postulation that TANGO2 protein plays a role in the transport of Golgi membranes to the ER^4,6^. However, studies on patient fibroblasts lacking TANGO2 protein found no indication of Golgi-ER fusion or reduced Golgi volume^1^. Reports of the size of Golgi membranes in patient-derived fibroblasts have been inconsistent, with contradictory report of changes in Golgi organization or size between patients and controls^1,3,4,7^. However, additional studies have suggested a possible impairment in retrograde ER-Golgi trafficking^8^. Of particular interest is the finding that while ER-to-Golgi transport is significantly delayed in TANGO2 deficient patients, the production of TANGO2 protein in these patient cells can rescue the transport process^7^. Taken together, these findings suggest that TANGO2 does in fact play a significant role in ER-Golgi trafficking without a notable impact on Golgi morphology.

These hypotheses notwithstanding, demonstration of the localization of the TANGO2 protein has been elusive, with some studies suggesting that it is present both in the cytosol and Golgi membranes^1,6^. However, the clinical phenotypes associated with TANGO2-related disease particularly the metabolic findings, are typical of a disorder of mitochondrial energy metabolism, and the protein product of mouse *Tango2* (T10), the mouse ortholog of *TANGO2*, was reported to be mitochondrially localized^4,9^. T10 protein has also been observed to be expressed in mitochondria-enriched fractions of mouse brain lysates^4^. Assessing TANGO2 protein localization in humans has proven difficult due to the lack of a commercial antibody suitable for immunofluorescent imaging, and conflicting information on the presence of a mitochondrial targeting signal^1,2,7^. In one study, GFP-fusion proteins expressed in HeLa cells did not identify co-localization of TANGO2 with a known mitochondrial marker, while another found significant levels of TANGO2 protein in mitochondrial fractions of HeLa cells expressing similarly tagged TANGO2 protein^1,7^.

Regardless of TANGO2 protein localization, significant changes in mitochondria have been demonstrated in patient derived fibroblasts. Proteomic analysis identified significant changes in proteins involved in the ER-Golgi network, fatty acid oxidation (FAO), and amino acid metabolism, including a decrease in the mitochondrial carnitine/acylcarnitine carrier protein (MCAT)^8^. Confocal microscopy has demonstrated significant alterations in mitochondrial morphology in patient fibroblasts, but not in primary myoblasts^2,7^. Respiratory chain enzyme activity, particularly complexes 1 and 2, have been shown to be mildly reduced in some patients; however, flux through the FAO pathway has been reported to be both normal and reduced^1,2,4^. These findings implicate the role of mitochondrial processes in disease presentation, yet the importance of these findings to the broader disease mechanism remains unclear. Therefore, we sought to characterize mitochondrial function in patients with TANGO2-related disorder to further elucidate the pathogenic mechanism of the disease and resolve the contradictions present in the current literature.

## Materials and methods

### Study design

This study utilized skin-derived fibroblasts from patients with identified mutations in the *TANGO2* gene and was approved by the University of Pittsburgh IRB (protocols #PRO19040093 and #PRO19030195). Informed consent was obtained. For underaged participants, parental informed consent was obtained. Skin fibroblasts (ATCC, PCS-201-012) and (Coriell, GM08399), derived from 40 years old healthy female and 19 years old healthy female respectively, were used in this study as controls. All research studies were performed in accordance with relevant guidelines/regulations.

### Case selection

Patient P1 has been followed clinically at the Division of Genetic and Genomic Medicine, UPMC Children’s Hospital of Pittsburgh and was recruited into this research study based on her clinical whole exome sequencing (WES), which revealed homozygous *TANGO2* gene exons 3–9 deletion. Her brother, Individual P2, was subsequently recruited to the study post-mortem as he was also found to have the same deletion as his sister, P1. Patient P3 was diagnosed and followed by Dr. Haas at University of California, San Diego (Table 1).

**Table 1:** Clinical and genetic findings of TANGO2 deficient patients.

	P1	P2	P3
Age of presentation	6 months	Unknown	18 months
Current age	14 years	Deceased at age 6 years	7 years
Age at diagnosis	~ 10 years old	After death	2 ½ years
Gender	Female	Male	Female
Ethnicity	European	European	European
Mutation	Homozygous exons 3–9 deletion (NM_152906.5)	Homozygous exons 3–9 deletion (NM_152906.5)	Compound heterozygous for c.605 + 1G > A splice site variant and exons 3–9 deletion (NM_152906.5)
Other genetic findings	Heterozygous pathogenic c.610delC variant on exon 7 of *CACNB4* gene (NM_000726)	Heterozygous pathogenic c.610delC variant on exon 7 of *CACNB4* gene (NM_000726)	None
Head circumference	Less than 3rd percentile	N/A	23rd percentile
Rhabdomyolysis	No	N/A	Yes
Seizures	Yes	Yes	Yes
Cardiac findings/arrythmias	Nonspecific T wave abnormality	Cardiac enlargement	Slightly low EF (47%)
Developmental delay	Yes (Severe)	Yes (severe)	Yes (mild)
Hypothyroidism	No	N/A	N/A
Ophthalmologic findings	Nystagmus, strabismus, alternating exotropia, myopia, astigmatism, and optic disk pallor	N/A	Minimal divergent strabismus, right eye variable exophoria and variable dysconjugate gaze
Hypoglycemia	No	N/A	Yes
Elevated CPK	Yes (mild)	Yes	Yes
Hyperammonemia	Yes (mild)	N/A	No
Lactic acidemia	No	Yes	Yes
Metabolic acidosis	No	Yes	Yes
Acylcarnitine profile	Normal	N/A	Abnormal*
Urine organic acids	Normal	N/A	Abnormal**
Plasma amino acids	Normal	N/A	Normal

Metabolic findings documented during acute illness/metabolic crisis. N/A: Not available.

*Slight elevation of C0, C2 and C3DC with elevated ratios due to low normal C8:1.

**Elevated lactate, 2-OH Butyric acid, 3-OH Butyric acid, 3-OH Isovaleric acid, 3-OH valeric acid, 5-OH hexanoic acid, glutaric acid, adipic acid and pyruvic acid.

### Whole exome/genome sequencing

WES for P1 and P2 was performed at Baylor Miraca Genetics Laboratories, Houston, TX, using DNA extracted from blood sample (P1) and skin fibroblasts (P2). WES identified homozygous pathogenic deletions of exons 3–9 of the *TANGO2* gene in both P1 and P2.Whole genome sequencing (WGS) with Sanger confirmation for P3 was completed by Rady Children’s Institute of Genomic Medicine. WGS identified compound heterozygosity for a maternally inherited 3–9 exon *TANGO2* deletion and a paternally inherited c.605 + 1G > A splice site variant in P3.

### Cell culture

Patient and control fibroblasts were grown in Dulbecco’s Modified Eagle Medium (DMEM) containing glucose (4.5 g/l) and supplemented with 10% fetal bovine serum, 2 mM glutamine, 100 IU penicillin, 100 μg/ml streptomycin, and 100 μg/ml normocin (InvivoGen, San Diego, CA, USA) at 37 °C, 5% (v/v) CO_2_. For glucose-free conditions, cells were incubated in the same medium devoid of glucose. Cells were tested regularly for mycoplasma contamination using a PCR Mycoplasma Detection Kit (Supplementary Table S3). Cells were used at passage number < 12.

### Western blot analysis

Western blotting was performed on whole cell samples from each patient cell line and a control line. Cell pellets of fibroblasts were resuspended in 4X their weight of sterile water and sonicated twice for 10 s each in a probe sonicator. Protein concentrations were determined using the DC™ Protein Assay kit (Bio-Rad Laboratories, Hercules, CA). The samples were then treated with 4X Laemmili buffer with β-mercaptoethanol and boiled to 95 °C for 8 min. Finally, samples were spun at 20,000xg for 20 min to pellet out any non-protein associated membranes that could otherwise interfere with the gel electrophoresis. Twenty five µg of protein was loaded onto a 4–15% gradient SDS-PAGE gel (Bio-Rad). Following electrophoresis, the gel was blotted onto a nitrocellulose membrane, blocked in 10% skimmed milk for 1 h at room temperature, and incubated in primary antibody overnight at 4 ˚C. Antibodies used in this study are listed in supplemental Table S1. They include anti-very long chain acyl-CoA dehydrogenase (VLCAD), generated in rabbits using recombinant proteins made in our lab by Cocalico (Cocalico Biologics, Stevens, PA) (1:1,000), as well as commercial antibodies anti-human C22orf25 (1:600), anti-medium chain acyl-CoA dehydrogenase (MCAD) (1:10,000), anti-isovaleric acid-CoA dehydrogenase (IVD) (1:1,000), and anti-electron-transferring-flavoprotein dehydrogenase (ETFDH) (1:300). Additionally, mitochondrial markers anti-heat shock protein 60 (Hsp60) (1:1,000), anti-adenylate kinase 2 (AK2) (1:1000), and anti-TOMM20 (1:10,000) were used. To assess mitochondrial fusion and fission functionality and ER stress, the same procedure was used except the gel was blotted onto a PVDF membrane and primary antibodies used were anti-mitofusin 1 (MFN1) (1:500), anti-mitofusin 2 (MFN2) (1:300), anti-optic atrophy type 1 (OPA1) (1:1000), anti-dynamin related protein 1 (DRP1) (1:500), anti-inositol 1,4,5-trisphosphate receptor (IP3R) (1:50), anti-glucose-related protein 75 (GRP75) (1:250), anti-glucose-related protein 78 (GRP78) (1:250), and anti-DNA damage inducible transcript 3 (DDIT3) (1:250). To assess the presence of the respiratory chain complexes, the above procedure was followed but samples were heated only to 42˚C for 10 min, 20 µg of protein was loaded onto an SDS-PAGE gel, and the gel was transferred to a PVDF membrane. A total oxidative phosphorylation (OXPHOS) antibody cocktail was used (1:300) along with anti-MTCO1 primary antibody (1:2000). These incubations were followed by corresponding HRP-conjugated secondary antibody. Primary purified mouse anti-GAPDH monoclonal antibody (1:25,000) was used as a loading control (Supplementary Table S1, S3).

### Mitochondrial isolation

Mitochondrial lysates were prepared from frozen cell pellets as described previously^10^. The isolated mitochondria were used for western blotting as above^11^. Thirty µg of protein was loaded onto an SDS-PAGE gel and blotted onto a nitrocellulose membrane following electrophoresis. Primary anti-human C22orf25 polyclonal antibody (1:75) was used followed by corresponding HRP-conjugated secondary antibody. HRP-conjugated mouse β-actin monoclonal antibody (1:5000) and anti-heat shock protein 60 (Hsp60) (1:1000) were used as a loading controls (Supplementary Table S1).

### Measurement of mitochondrial respiration

Mitochondrial respiration was monitored using a Seahorse XFe96 Extracellular Flux Analyzer (Agilent, Santa Clara, CA). OXPHOS was monitored by measuring oxygen consumption rate (OCR). Cells were grown in 2 T75 flasks in complete DMEM. Twenty-four hours prior to assaying, media in one flask per cell line was changed to glucose-free DMEM. Cells were harvested and seeded in 96-well Seahorse cell culture microplates precoated in poly-D lysine in growth media at a density of 80,000 cells per well (n = 8 replicates per cell line) on the morning of the assay. The incubation conditions and subsequent addition of oligomycin, carbonyl cyanide 4-(trifluoromethoxy) phenylhydrazone (FCCP), and Rotenone/antimycin A (Seahorse XF Cell Mito Stress Test Kit, Supplementary Table S3) were performed according to manufacturer guidelines. OCR data was normalized to protein and reported as pmol/min/μg protein.

### Measurement of ATP content and production

Total cellular ATP content was measured using the ATPlite™ bioluminescence assay according to the manufacturer’s instructions (Supplementary Table S3). The day before the assay, approximately 5,000 cells were seeded in 4 replicates in 96-well plates. The luminescence was measured in a SpectraMax® i3x Platform multi-mode microplate reader system (Molecular Devices, LLC, Sunnyvale, CA). Data were reported in μmol and was normalized to protein and reported as μmol/μg/μl protein. ATP production rate was measured using the Seahorse XF Real-Time ATP Rate Assay Kit on a Seahorse XFe96 Extracellular Flux Analyzer (Seahorse Bioscience, Billerica, MA). Cells were seeded in 96-well Seahorse tissue culture microplates pre-coated in poly-D-lysine at a cell density of 60,000 cells/well the day before doing the assay. 16 wells of each cell lines were seeded in complete DMEM. After seeding, the cells were allowed to rest at room temperature for 1 hour to prevent edging effects and then moved to a 37 °C, 5% CO_2,_ incubator for three hours. Media in eight wells with each cell line were changed to glucose-free DMEM and cells incubated overnight to mimic cellular stress. The assay then was performed with successive addition of oligomycin and Rotenone/antimycin A to determine the ATP production rate from mitochondrial respiration and glycolysis, respectively (Supplementary Table S3).

### Measurement of superoxide production and mitochondrial mass

Confluent T182 flasks of patient cell lines were incubated for 24 h in either complete or glucose-free DMEM. Then, cell suspension containing 300,000 cells/ml were incubated for 20 min at 37 °C with 5 µM MitoSOX Red (Invitrogen, Grand Island, NY) and 150 nM Mitotracker Green (Invitrogen). After incubation, 50,000 cells were analyzed per cell line in a Becton Dickinson FACSAria II flow cytometer (BD Biosciences, San Jose, CA).

### Measurement of mitochondrial membrane potential

Mitochondrial membrane potential was measured using live-cell imaging following staining with the JC-1 dye (Invitrogen). Cultured control and patient fibroblasts were seeded onto 35 mm MatTek dishes (MatTek, Ashland, MA) at a density of 40,000 cells in complete DMEM. On the day of the assay, the media was removed, and cells were washed with phosphate buffered saline (PBS) containing calcium. One µl of the JC-1 dye, diluted to 1 mg/ml in DMSO, was mixed in 1 ml PBS, added to the dish, and incubated for 30 min at 37 °C, 5% (v/v) CO_2_. After incubation, the cells were rinsed with PBS, complete DMEM was added, and the dish was placed into a closed, thermo-controlled (37 °C) stage top incubator above the motorized stage of an inverted Nikon TiE fluorescent microscope equipped with a 60 × optic Nikon, CFI Plan Fluor, NA 1.4 (Nikon Inc. Melville, NY). The JC-1 dye was excited using a diode-pumped light engine (SPECTRA X, Lumencor) and detected using an ORCA-Flash 4.0 sCMOS camera (Hamamatsu) and excitation and emission filters from Chroma Technology Corp (Bellows Falls, VT). Data were collected on approximately 10–20 cells per stage position, with 10–15 stage positions per condition, were analyzed using NIS Elements software (Nikon Inc.). The ratio of red:green (hyperpolarized:depolarized) fluorescence was calculated.

### Fatty acid oxidation (FAO) flux assay

Control and patient fibroblast cells were transferred from T175 flasks onto Corning brand 6-well plates with a seeding density of 350,000 cells per well in triplicates for the assay and duplicates to determine protein concentration, and incubated for 24 h in a 37 °C. The plates were then incubated for 2 h in a mixture of glucose-free media supplemented with L-carnitine and 100 µM tritiated [9,10-^3^H]oleate (protein concentration plates were incubated with mixture of glucose-free media supplemented with L-carnitine only) respectively (Supplementary Table S3). Flux through the FAO pathway was quantified by tritium release from [^3^H]oleic acid conjugated to fatty acid-free albumin, as previously described^10,12^. Reaction mixture was quenched with water, transferred to AG 1-X8 Resin column (Bio-Rad Laboratories) and eluted into scintillation vials. 10 ml of scintillation fluid was then added to each vial, shaken, and allowed to incubate at room temperature overnight before counting on a Beckman Coulter LS 6500 MultiPurpose Scintillation Counter (Beckman Coulter, Brea, CA). The oxidation rate was expressed as nmol 3H released/h/mg protein.

### Quantification of mRNA expression

Cells from four T25 flasks per cell line were grown to 100% confluency, harvested, and stored at − 80 °C. RNA was extracted using the RNeasy Mini Kit (Qiagen, Valencia, CA), RNA concentration measured via NanoDrop (Thermo Scientific, Waltham, MA, USA), and the resulting RNA used to synthesize cDNA using the SuperScript™ IV VILO™ Master Mix (Invitrogen). The cDNA was diluted to a final concentration 2.5 ng/μl in nuclease-free water. In a 96-well clear PCR plate, 5 ng of cDNA was added into each well in technical duplicates and biological triplicates. qPCR analysis was performed on a Bio-Rad Real-Time PCR system (Bio-Rad Laboratories) using PowerUp™ SYBR™ Green Master Mix (Thermofisher Scientific, Waltham, MA) and 10 mM primers for estimating the presence of VLCAD, MCAD, IVD, ETFDH, UQCRC2, TOMM20, and GAPDH. The average of technical replicates for each protein assayed was normalized to GAPDH run on the same plate and analyzed using the Livak method.

### Measurement of mitochondrial DNA copy number

Mitochondrial DNA copy number was quantified by a Taqman probe-based droplet-digital PCR (ddPCR) assay run on an automated droplet generator and QX200 reader (Bio-Rad Laboratories) using optimized oligonucleotide primers and PrimeTime qPCR probes (Integrated DNA Technolgies, Coralville, IA). Total DNA (nuclear and mitochondrial genome) was isolated from fibroblast cell pellets with proteinase K (Invitrogen) digestion followed by phenol–chloroform extraction and ethanol precipitation. DNA quality was checked by 0.8% agarose gel electrophoresis and quantified by broad range DNA Qubit (Fisher Scientific, Waltham, MA). Prior to ddPCR genomic DNA was digested with EcoRI (New England Biolabs, Ipswich, MA) and diluted to 10 ng/ul. Two different multiplex Taqman ddPCR assays were run with alternate pairs of mitochondrial DNA target and nuclear DNA reference amplicons (mtND1/B2M or mtCYB/RPP30) (Supplemental Table S2). Triplicate reactions for each sample of either 2 µl undiluted (20 ng total) or 1:100 diluted (0.2 ng total) were run and the mtDNA copy number inferred by the ratio of concentration (copies per µl) of the 1:100 diluted to diploid nuclear reference in the undiluted multiplied by 200. The standard deviation of each sample was calculated from the triplicate Taqman assays and statistical comparison to a single control fibroblast control was made by a 2-way student’s T-test.

### Transmission electron microscopy (TEM)

The specimens were fixed in cold 2.5% glutaraldehyde in PBS, pH 7.3. The specimens were rinsed in PBS, post-fixed in 1% osmium tetroxide with 1% potassium ferricyanide, dehydrated through a graded series of ethanol (30–90%) and embedded in Poly/Bed® 812 (Luft formulations). Semi-thin (300 nm) sections were cut on a Leica Reichart Ultracut (Leica Microsystems, Buffalo Grove, IL), stained with 0.5% Toluidine Blue in 1% sodium borate and examined under the light microscope. Ultrathin Sects. (65 nm) were stained with 2% uranyl acetate and Reynold’s lead citrate and examined on JEOL 1400 transmission electron microscope (JEOL Peabody, MA) (NIH grant #1S10RR016236-01, Simon Watkins) with a side mount AMT 2 k digital camera (Advanced Microscopy Techniques, Danvers, MA).

### Measurement of mitochondrial protein expression

Patient and control fibroblasts were seeded on tissue culture-treated glass coverslips at a density of 25,000 cells per coverslip and allowed to grow overnight at 37 °C in a 5% CO_2_, 95% humidity incubator. Cells were fixed, permeabilized, and blocked as described in previous publications^13^. Primary antibodies used include anti-VLCAD (1:1,000), anti-MCAD (1:100), anti-IVD (1:500), anti-ETFDH (1:100), anti-TOMM20 (1:100). Primary antibody incubation was followed by incubation in appropriate Alexa Fluor-488 secondary antibody (1:1000) for 1 h at room temperature, then a 2-min incubation in 1 μl/ml Hoescht stain. Coverslips were then mounted and imaged on a Nikon A1 Confocal microscope at 60X magnification using NIS Elements Software. For anti-TOMM20 images, large scale (3 × 3) images were collected from 3 separate slides per cell line and were analyzed using NIS Elements software.

### Statistics

Statistical significance was assessed by performing the one-way nonparametric ANOVA with Tukey’s post-hoc test for pairwise comparisons or unpaired Student’s T-test using GraphPad Prism version 8.00 for Mac, GraphPad Software (La Jolla, California, USA, www.graphpad.com). A *p* value < 0.05 was considered significant. *p* value: **** ≤ 0.0001; *** ≤ 0.001; ** ≤ 0.01; * ≤ 0.05; ns > 0.05. Error bars represent standard error.

## Results

### Localization of TANGO2 protein to mitochondria

**Figure 1:**
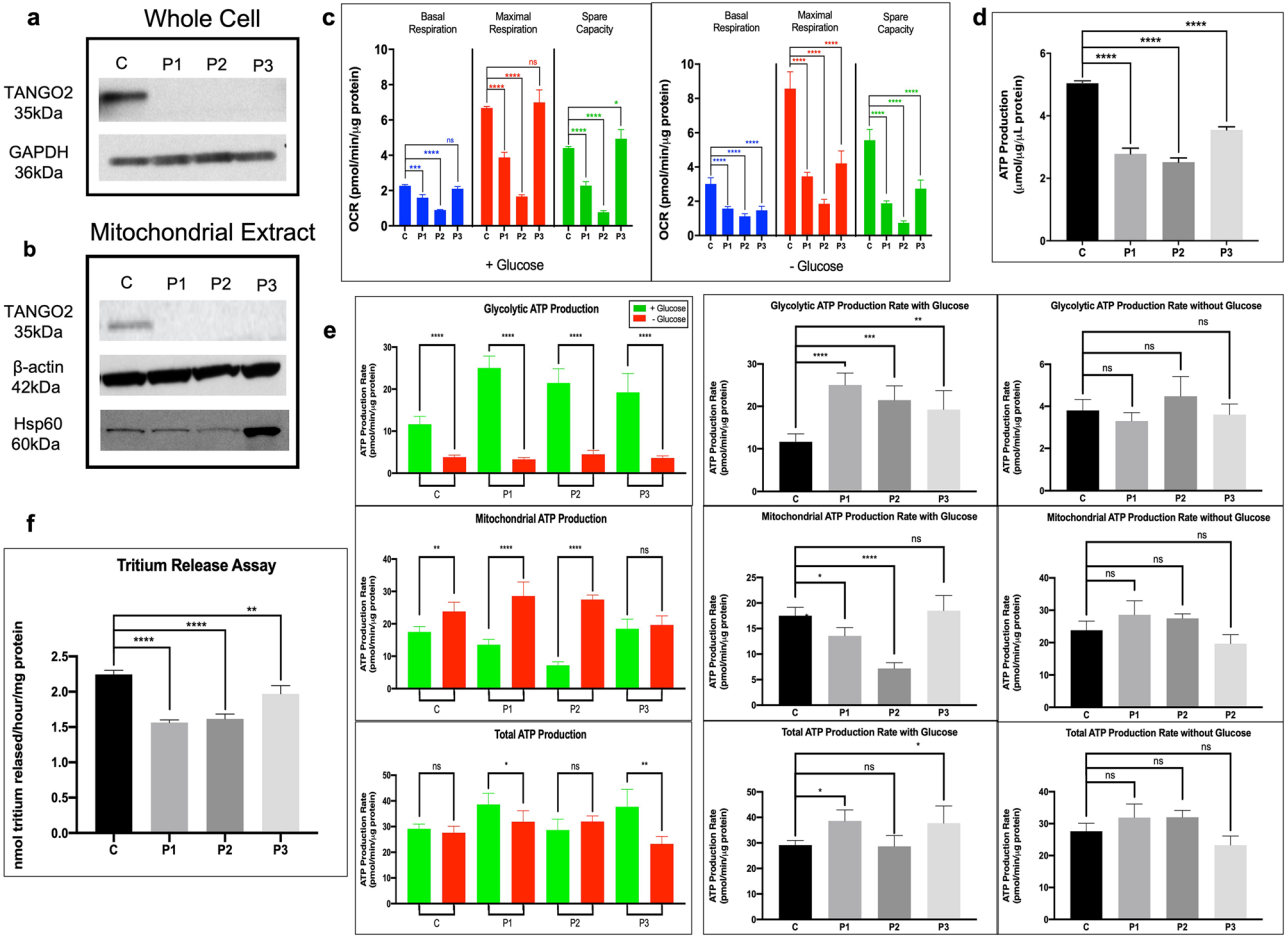
Presence of TANGO2 protein in mitochondria and loss of mitochondrial function through cellular respiration, ATP production, and fatty acid oxidation. (**a**) Whole cell extracts from fibroblasts of three patients with TANGO2-related disease and a control were analyzed for the presence of TANGO2 antigen by SDS-PAGE western blotting with anti-TANGO2 and anti-GAPDH antibodies. Twenty-five μg protein was loaded for all. Cropped images shown, full-length blots are presented in Supplementary Fig. S1. (**b**) Mitochondrial extracts from fibroblasts of three patients with TANGO2 disorder and a control were analyzed for the presence of TANGO2 antigen by SDS-PAGE western blotting with anti-TANGO2, anti-β-actin and anti-Hsp60 antibodies. Thirty μg protein was loaded for all. Cropped images shown, full-length blots are presented in Supplementary Fig. S2. (**c**) Oxygen Consumption Rate (OCR) data was measured with a Seahorse XFe96 Extracellular Flux Analyzer and presented as basal respiration, maximal respiration, and spare capacity for both conditions: with and without glucose. With glucose, only P1 and P2 show reduced oxygen consumption of all 3 measures relative to control while when under stress, in the no glucose condition, all patients show reduced OCR in all measures. Data presented as pmol/min/μg protein. (**d**) ATP content monitored using the ATPlite™ bioluminescence assay showing significantly reduced ATP production in all patients relative to control. (**e**) ATP Production Rate was measured with a Seahorse XFe96 Extracellular Flux Analyzer to calculate distinct ATP production from glycolysis and mitochondrial respiration when incubated with and without glucose. Total ATP production rate was increased in P1 and P3 relative to control in normal conditions. Without glucose, the patients ATP production rates do not differ from control. (**f**) Tritium release assay performed with tritiated palmitate showing reduced palmitate metabolism through the β-oxidation pathway. *p* value: ****≤ 0.0001; ***≤ 0.001; **≤ 0.01; *≤ 0.05; ns > 0.05. Error bars represent standard error.

TANGO2 protein was easily identifiable in western blot analysis from whole cell (Fig. 1a) and mitochondrial extracts (Fig. 1b) of control fibroblasts, but no signal was detected with extracts of any of the patients’ fibroblasts. Note that the control TANGO2 signal was less intense on western blotting of mitochondrial extracts than in whole cell samples, suggesting that TANGO2 is also present outside of the mitochondria.

### Characterization of mitochondrial dysfunction in TANGO2 deficient patient fibroblasts

Mitochondrial respiration in patient fibroblasts measured as oxygen consumption rate (OCR) was abnormal compared to control fibroblasts both in the presence of and in the absence of glucose (Fig. 1c). In the presence of glucose, P1 and P2 had significantly lower OCR (basal respiration, maximal respiration, and spare respiratory capacity) values than the control, while P3’s OCR did not significantly differ from control. When incubated in glucose-free media prior to assaying, all patients, displayed reduced OCR relative to control (Fig. 1c).

ATP content was significantly decreased in all patients’ fibroblasts compared to control (Fig. 1d). This is consistent with a cellular energy imbalance and patients having significantly increased glycolytic ATP production rates, but decreased or comparable mitochondrial ATP production rates compared to controls when incubated with glucose (Fig. 1e). These findings suggest some degree of mitochondrial dysfunction in patient cells. When the cells were starved of glucose, neither glycolytic nor mitochondrial ATP production differed significantly from control. In P1 and P2 cells, total ATP production rate did not change with glucose supplementation, while P3 showed a significant drop in total ATP production rate in the absence of glucose compared to with glucose.

**Figure 2:**
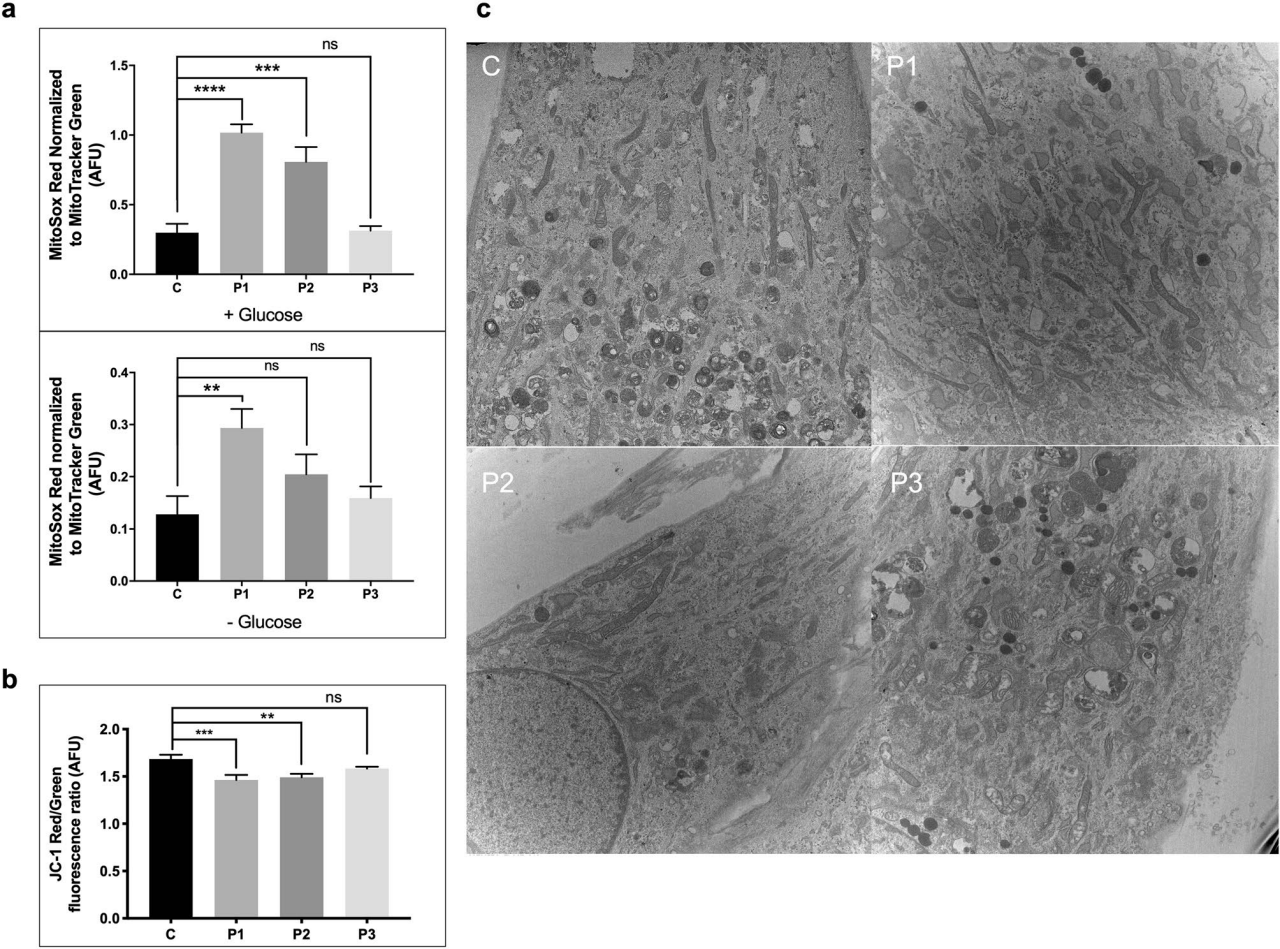
Assessment of mitochondrial damage. (**a**) Reactive oxygen species (ROS) production measured using MitoSox Red and normalized to mitochondrial mass as measured using MitoTracker Green, with and without glucose. ROS production is high in P1 and P2 relative to control in both conditions. (**b**) Mitochondrial membrane potential as measured using live-cell imaging of JC-1 dye, showing reduced Red/Green (hyperpolarized/depolarized) fluorescence ratio, and therefore depolarized membrane potential in P1 and P2 relative to control. (**c**) Transmission electron microscopy images at 10,000X magnification from patients and control, showing no visual differences in the fine structure of patient mitochondria. *p* value: ****≤ 0.0001; ***≤ 0.001; **≤ 0.01; *≤ 0.05; ns > 0.05. Error bars represent standard error.

All patient cell lines demonstrated significantly reduced flux through mitochondrial FAO measured with tritiated oleate (C18) as substrate compared to control (Fig. 1f). Additionally, superoxide production in P1 and P2 cells was increased compared to control cells with and without glucose while it was not in P3 (Fig. 2a). Mitochondrial membrane potential was depolarized in P1 and P2, but not in P3 (Fig. 2b).

### Mitochondrial morphology and dynamics

**Figure 3:**
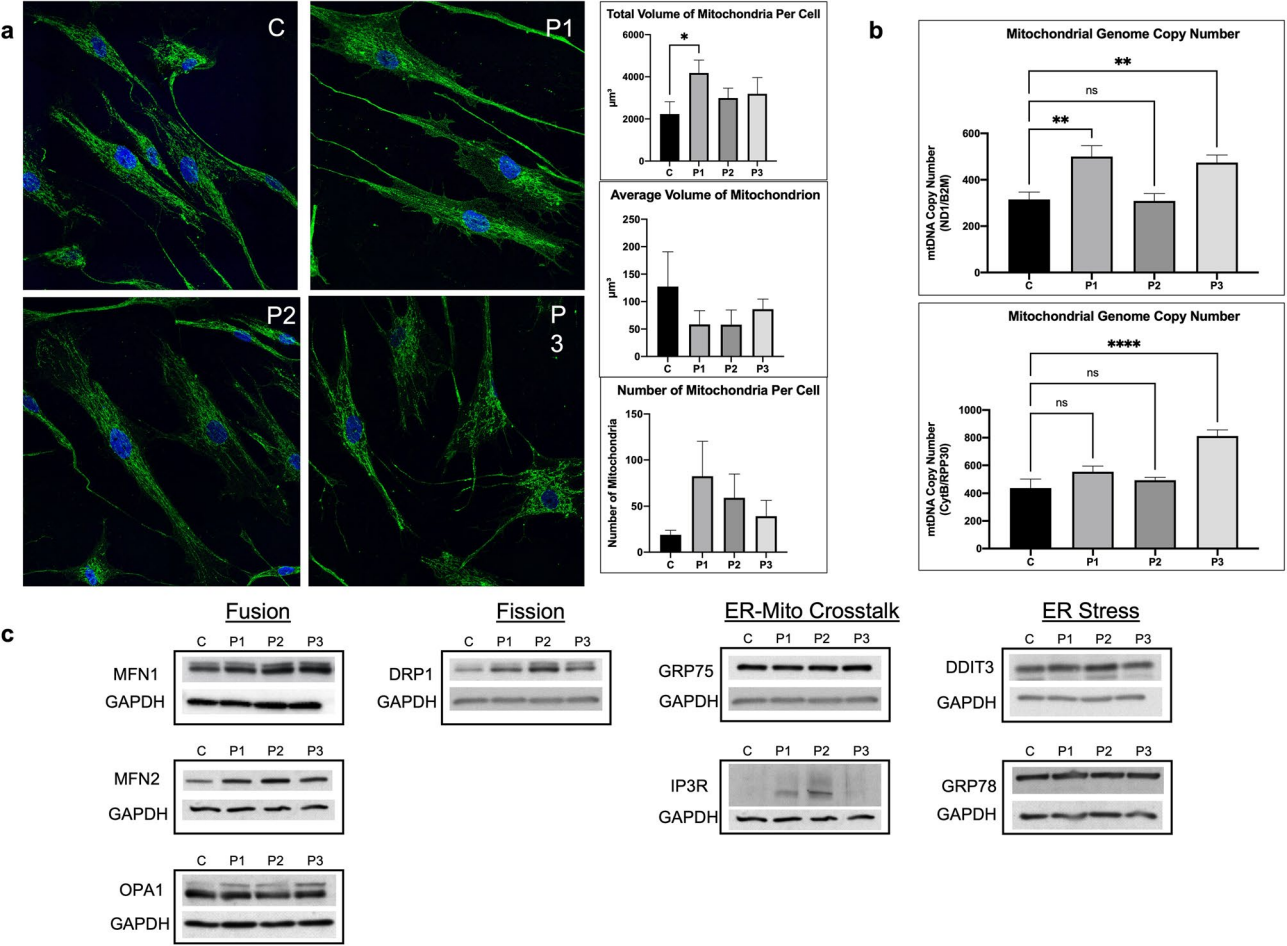
Evaluation of mitochondrial dynamics. (**a**) Immunofluorescent staining of fibroblasts from TANGO2 patients and a control. TOMM20 antigen was visualized with green fluorescently tagged antibodies and analyzed using the Nikon NIS-Elements software for mitochondrial number and volume. Nuclei were visualized with Hoescht-Blue stain. Large scale (3 × 3) images were taken at 60 × magnification. Images presented are cropped for easier visualization. Patients were observed to have significantly increased mitochondrial number and decreased volume per mitochondrion, suggesting more fractured mitochondria relative to control. (**b**) Digital droplet PCR was used to measure mitochondrial DNA copy number using 2 mitochondrial genes: ND1 and CytB, and 2 nuclear genes: B2M and RPP30. Data is presented as the ratio of mitochondrial DNA copy number:nuclear DNA copy number. (**c**) Mitochondrial fission and fusion dynamics, as well as the ER-mitochondrial crosstalk axis and relative ER stress were assessed via SDS-PAGE western blotting of whole cell extracts from fibroblasts from patients and control. A variety of antigens known to be involved in these pathways were used. Twenty-five μg protein was loaded for all. Cropped images shown, full-length blots are presented in Supplementary Figs. S3–S6. *p* value: ****≤ 0.0001; ***≤ 0.001; **≤ 0.01; *≤ 0.05; ns > 0.05. Error bars represent standard error.

To examine mitochondrial structural integrity and dynamics more broadly, we utilized morphological evaluation, quantitated mitochondrial abundance and size, and quantified mtDNA content using a variety of different techniques. TEM images showed no changes in the fine structure of the mitochondria between control and patients (Fig. 2c). Immunofluorescence of TOMM20, an inner mitochondrial membrane protein, was visually similar in patient and control fibroblasts. However, analysis of total mitochondrial volume per cell by immunofluorescence demonstrated increased total mitochondrial volume and number in patient cells compared to control along with reduced volume per mitochondrion, suggesting that the patient mitochondria are more fragmented compared to control (Fig. 3a). This is consistent with western blotting of fission-related DRP1, showing increased mitochondrial fission in patients (Fig. 3c). Similarly, ddPCR showed increased or similar mitochondrial DNA copy number in patients when compared to control (Fig. 3b). However, the increase in mitochondrial DNA copy number is smaller than the increase in the number of mitochondria per cell, suggesting that patients’ mitochondria contain fewer copies of mtDNA compared to control. In addition, markers for mitochondrial fission and fusion as well as for changes in ER-mitochondrial crosstalk and ER stress were examined^14^. No difference was seen in the protein level between patients and control of the fusion proteins MFN1, MFN2 and OPA1, but the fission-related DRP1 was increased in patients, particularly P2. ER stress proteins including DDIT3 and Grp78 were unchanged in patients’ cells compared to control, while ER-mitochondrial crosstalk proteins GRP75 and IP3R levels were inconsistent, with no difference seen in the level of GRP75 but an increase in the IP3R signal in patient cells (Fig. 3c).

### Expression analysis of selected mitochondrial proteins

**Figure 4:**
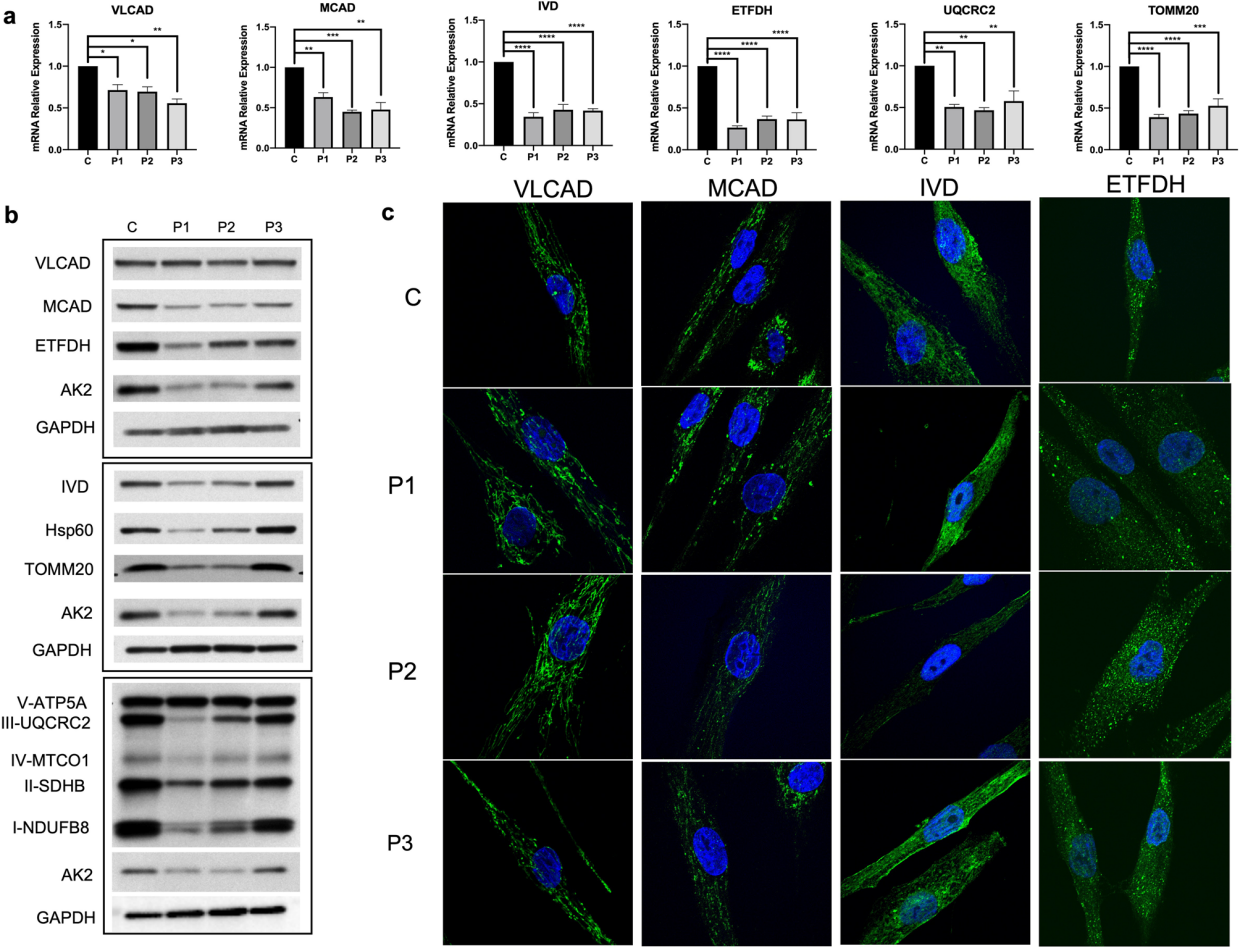
Characterization of FAO and respiratory chain complexes. (**a**) mRNA expression of various mitochondrial proteins, from different sections of the mitochondria, was analyzed using qPCR in patient and control fibroblasts. qPCR revealed reduced mRNA expression of all proteins tested compared to control. (**b**) Mitochondrial proteins involved in various pathways of mitochondrial respiration including FAO and respiratory chain were analyzed via SDS-PAGE western blotting with antigens for the corresponding proteins. Mitochondrial and cytosolic loading controls were used: anti-AK2 and anti-GAPDH antigens, respectively. Twenty-five μg protein was loaded for the anti-VLCAD, anti-MCAD, anti-ETFDH, anti-IVD, and anti-Hsp60 blots. Twenty μg protein was loaded for the respiratory chain complexes. Cropped images shown, full-length blots are presented in Supplementary Figs. S7–S9. (**c**) Immunofluorescent staining of fibroblasts from patients and a control using antigens for mitochondrial proteins VLCAD, MCAD, IVD, and ETFDH. These proteins were visualized with green fluorescently tagged antibodies to evaluate the presence of these proteins and nuclei were visualized with Hoescht-Blue stain. Images were taken at 60X magnification. *p* value: ****≤ 0.0001; ***≤ 0.001; **≤ 0.01; *≤ 0.05; ns > 0.05. Error bars represent standard error.

Expression of selected genes for nuclear encoded mitochondrial protein genes, including some involved in mitochondrial FAO and OXPHOS was measured with real time PCR (qPCR) and protein levels were estimated by western blotting or immunofluorescence staining. When normalized to *GAPDH* as a control, mRNA expression of *ACADVL* (VLCAD), *ACADM* (MCAD), *IVD*, *ETFDH*, *TOMM20* and *UQCRC2* was decreased in all TANGO2 patients (Fig. 4a). These changes were mirrored by protein levels as seen with western blotting except for VLCAD, which was unchanged in patient cells (Fig. 4b). On immunofluorescent staining, VLCAD protein levels also appeared to be similar between control and patient samples, MCAD, IVD and ETFDH signals were reduced in patient fibroblasts (Fig. 4c).

## Discussion

TANGO2 deficiency is an autosomal recessive disorder that has been associated with phenotypic and metabolic variability in patients and patient derived samples^5^. While mitochondrial energy dysfunction has been postulated, the precise function of TANGO2 protein and the pathophysiology of the disease remains unclear. Our studies are consistent with the conclusion that the TANGO2 protein is at least partially localized to mitochondria in cells as observed in mitochondrial lysates of control fibroblasts and has considerable effects on mitochondrial bioenergetics and structure^7,15,16^.

Mitochondria provide the bulk of cellular ATP under most circumstances through the combined function of FAO, Krebs cycle, and OXPHOS^17,18^. Our studies demonstrated changes in FAO and OXPHOS, with alterations in mitochondrial respiration (OCR), ATP production, FAO flux, and expression of several proteins involved in both pathways. All of the patient fibroblasts demonstrated decreased mitochondrial respiration under stress (glucose-free media). However, only patients P1 and P2 who are siblings with the homozygous 3–9 exon deletion and who are clinically more affected than P3, exhibited decreased mitochondrial respiration without stress (in media containing glucose) as evident in mitochondrial basal, maximal and spare capacity. FAO flux was decreased in all patients, supporting previous conjecture that *TANGO2* mutations result in a functional defect in mitochondrial β-oxidation^4,8^. Our findings specifically identify a defect in long chain FAO, while the metabolism of medium and short chain fatty acids remains to be investigated. In addition, alterations in protein level and mRNA expression of a variety of mitochondrial proteins demonstrate that multiple mitochondrial sub-compartments are affected in patient cells, including both the inner (VLCAD, ETFDH) and outer (TOMM20) mitochondrial membranes, the mitochondrial matrix (MCAD, IVD), and the respiratory chain (all mitochondrial complexes including UQCRC2, a subunit of mitochondrial complex III). Here, multiple respiratory chain complex proteins were affected, including complexes I, II, III and IV, in addition to MCAD, IVD, ETFDH and TOMM20, AK2 and HSP60 (mitochondrial biomarkers). Reduction in expression of genes involved in both implicate broad mitochondrial dysfunction with secondary alteration of coordinated expression.

With defects in both FAO and OXPHOS, TANGO2 deficient patients have a greater dependency on glycolysis to meet energy needs, consistent with the observed decreased net steady state ATP levels, reduced production through OXPHOS, and increased ATP production through glycolysis as seen in patient cells. Measurement of ATP content and production provides a detailed look at where in the pathway ATP production is disrupted. The luminescence assay provides net ATP content measured at a single point in time, which is decreased in patients, while the ATPrate assay measures ATP production per minute. The lack of difference in total ATP production with and without glucose in the growth media, along with the reduced ATP content, suggests that while the cells are able to maintain ATP production rate under stress, TANGO2 patients have an increased ATP need relative to control. Together these findings suggest that while patient cells are able to maintain similar ATP production rates to the control at baseline and under stress by increasing ATP production from glycolysis and increasing mitochondrial number, these patients have an increased ATP need leading to a net ATP deficit. This likely explains at least in part their tendency to show clinical exacerbations during times of physiologic stress such as intercurrent illnesses. Of note, pateint P3 had the least affected bioenergetics, consistent with her milder presentation than P1 and P2. Additionally, the reduced mitochondrial ATP production observed in the cells grown in glucose-supplemented media correlates with the depolarized mitochondrial membrane potential in P1 and P2. Reduced proton pump capability of the electron transport chain sequesters protons in the mitochondrial matrix, depolarizing the cell and resulting in reduced mitochondrial ATP production.

Mitochondria are highly dynamic organelles that constantly undergo fission and fusion in order to maintain mitochondrial shape, structure and function^19,20^. Therefore, we studied a number of markers of mitochondrial size and shape. This included the content of proteins involved in mitochondrial dynamics including DRP1 (fission) and MFN1, MFN2, OPA1 (fusion) in addition to TOMM20, a mitochondrial marker, immunofluorescence, and a ddPCR assay of mtDNA copy number. Our findings are consistent with an increased mitochondrial content in patient cells as evidenced by increased DRP1 protein (a marker for fission), increased number of mitochondria per cell, and decreased mitochondrial size (as determined by TOMM20 immunofluorescence). Additionally, while ddPCR identified an increased mtDNA copy number in patient cells compared to control, it was not sufficient to match the increase in mitochondrial abundance. In total, patient cells contain a higher number of mitochondria that are smaller in size, and each mitochondrion contains fewer copies of the mtDNA compared to control cells, indicative of mitochondria that are more fragmented, undergoing more fission than controls, and replicating mitochondrial DNA at a slower rate than control. In addition to the change in the mitochondrial bioenergetics and dynamics, ROS production was increased in cells from P1 and P2, and had a reduced mitochondrial membrane potential, findings consistent with mitochondrial damage related to the *TANGO2* mutations.

Finally, we investigated ER-mitochondrial crosstalk and ER stress in patient derived fibroblasts. ER-mitochondrial crosstalk through mitochondrial associated membranes (MAMs) is critical for maintenance of normal mitochondrial function^14,21,22^. Key proteins in this process include the ER Ca^2+^ channel IP3R and the mitochondrial chaperone GRP75^23^. We showed no change in GRP75 in patient cells; however, IP3R levels were increased in P1 and P2 derived fibroblasts. Thus, alteration in the mitochondrial ER crosstalk axis are likely, though additional studies are necessary to further examine the integrity of the complex formed by IP3R, GRP75 and two additional proteins. Of note, DDIT3 and GRP78, two ER stress markers, were not increased in the patient cell lines suggesting a more subtle defect in communication with mitochondria rather than global ER dysfunction. Notably, our studies did not address the role of ER-Golgi trafficking in TANGO2 deficiency pathophysiology, though the lack of a signal of global ER stress makes such dysfunction less likely. Ultimately, we hypothesize that the TANGO2 protein plays a critical role in integrating the physical connection between the ER-Golgi network and the mitochondria.

In conclusion, our data demonstrate a picture of cellular dysfunction that extends beyond the significant mitochondrial energy deficit in TANGO2 deficient cells and highlights the need to better understand the normal function of this protein before rational medical therapies can be developed. Given the difference in severity of the various cellular functions in patients with different phenotypes, this knowledge will allow greater insights into genotype/phenotype correlations in patients.

## Supplementary Information

Supplementary Information 1.

Supplementary Information 2.

## Data Availability

All materials used in this study are commercially available. All data are available upon request as mandated by NIH guidelines.
