# Supplementary material for: Mitochondrial dysfunction associated with TANGO2 deficiency
Source: Sci Rep. 2022 Feb 23;12:3045. doi: 10.1038/s41598-022-07076-9 (PMC8866466; doi:10.1038/s41598-022-07076-9)

Mitochondrial Dysfunction Associated with TANGO2 Deficiency

Paige Heiman[1], Al-Walid Mohsen[1,2], Anuradha Karunanidhi[1], Claudette St Croix[3], Simon Watkins[3], Erik Koppes[1], Richard Haas[4], Jerry Vockley[1,2], Lina Ghaloul-Gonzalez[1,2]*

[1]Division of Genetic and Genomic Medicine, Department of Pediatrics, University of Pittsburgh, Pittsburgh, PA, USA.

[2]Department of Human Genetics, Graduate School of Public Health, University of Pittsburgh, Pittsburgh, PA, USA

[3]Center for Biologic Imaging, Department of Cell Biology, University of Pittsburgh, Pittsburgh, PA, USA

[4]Departments of Neurosciences and Pediatrics, Division of Pediatric Neurology, University of California San Diego and Rady Children's Hospital-San Diego, San Diego, CA, USA

*To whom correspondence should be addressed: Lina.Gonzalez@chp.edu

Supplementary Information

Detailed Clinical Case Summaries

Individual P1 is 14-year-old female of European descent who first presented at 6 months of age with profound developmental delay, failure to thrive, seizures, alternating exotropia, dysphagia and generalized brain volume loss. She had an abnormal EEG displaying multifocal and diffuse waves and spikes, along with white matter changes and ventricular dilation on MRI. During hospitalizations with acute illnesses, P1 demonstrated slightly elevated CPK (highest 259 IU/L, NR: 30-170 IU/L) and slight elevation of ammonia in only one of multiple hospital admissions (highest 99 µmol/L; NR: 12-38µmol/L) with no lactic acidosis. Multiple plasma acylcarnitine profile studies over time were normal. The patient continues to have intractable epilepsy (1-4 seizures per day) status post vagal nerve stimulator (VNS) implantation in addition to treatment with multiple antiepileptic medications. She has spasticity, with contractures in the upper and lower extremities, and low postural tone kyphosis. She is wheelchair and g-tube dependent and has a left dysplastic acetabulum. She has recurrent aspiration and sialorrhea requiring BOTOX injection into the salivary glands and uses BiPAP nocturnally. At 9 years of age, WES revealed a homozygous pathogenic deletion of exons 3-9 of the *TANGO2* gene. In addition, a heterozygous c.610delC (p.Gln204Lysfs*3) pathogenic variant on exon 7 in the *CACNB4* gene was found. The latter variant is associated with autosomal dominant generalized idiopathic epilepsy. However, the clinically well father is also heterozygous for the *CACNB4* variant, suggesting incomplete penetrance. Both parents are heterozygous for the exon 3-9 deletion of the *TANGO2* gene. WES also detected two heterozygous variants of unknown clinical significance in *SLC13A5* and *ANKRD11*.

P2 is the older brother of individual P1. He died at age 6 years from a presumed mitochondrial disorder, but with no molecular diagnosis. P2 presented with profound global developmental delay, seizures, cardiac enlargement, and severe lactic acidosis. WES was performed post-mortem on DNA extracted from patient skin fibroblasts and showed homozygous deletion of exon 3-9 deletion of the *TANGO2* gene as well as heterozygosity of the *CACNB4* variant.

Individual P3 is a 7-year-old female who first presented at 18 months of age with mild global developmental delay, strabismus, and possible complex partial seizures. During acute illness, she had a metabolic decompensation with hypoglycemia (lowest 37 mg/dL; NR: 70-126 mg/dL), lactic acidemia (highest 6.3 mmol/L; NR: 0.5-2.2 mmol/L) and rhabdomyolysis (highest CK 60,700 U/L; NR: 22-198 U/L). WGS identified compound heterozygosity for a maternally inherited 3-9 exon *TANGO2* deletion and a paternally inherited c.605+1G>A splice site variant. She has responded well to supplementation with levocarnitine, CoQ10, riboflavin and vitamin B50 and at 4 ½ years of age could walk and stand on her own, talk using more than 200 words and phrases, and complete puzzles.

Supplementary Table S1. List of antibodies

	Application Used	Catalog Number	Company	Concentration
**Anti-C22orf25 (TANGO2)**	Western Blot	27846-1-AP	ProteinTech Group, Inc. Rosemont, IL, USA	1:600
**Anti-VLCAD**	Western Blot and IF	-	Lab-Made	1:1000
**Anti-MCAD**	Western Blot and IF	ab92461	Abcam Cambridge, MA, USA	1:10000 (western) 1:100 (IF)
**Anti-IVD**	Western Blot	TA501742	Origene Technologies, Inc. Rockville, MD, USA	1:1000
	IF	-	Lab-Made	1:500
**Anti-ETFDH**	Western Blot	ab131376	Abcam Cambridge, MA, USA	1:300
	IF	PA5-100143	Invitrogen Waltham, MA, USA	1:100
**Anti-MTCO1**	Western Blot and IF	ab14705	Abcam Cambridge, MA, USA	1:2000
**Total OXPHOS Human WB Antibody Cocktail**	Western Blot	ab110411	Abcam Cambridge, MA, USA	1:300
**Anti-Hsp60**	Western Blot	12165	Cell Signaling Technology Danvers, MA, USA	1:1000
**Anti-AK2**	Western Blot	ab157206	Abcam Cambridge, MA, USA	1:1000
**Anti-TOMM20**	Western Blot	ab186735	Abcam Cambridge, MA, USA	1:10000
	IF	PA5-52843	Invitrogen Waltham, MA, USA	1:100
**Anti-MFN1**	Western Blot	ab57602	Abcam Cambridge, MA, USA	1:500
**Anti-MFN2**	Western Blot	SC-100560	Santa Cruz Biotechnology, Inc. Dallas, Texas, USA	1:300
**Anti-OPA1**	Western Blot	ab119685	Abcam Cambridge, MA, USA	1:1000
**Anti-DRP1**	Western Blot	ab56788	Abcam Cambridge, MA, USA	1:500
**Anti-IP3R**	Western Blot	SC-377518	Santa Cruz Biotechnology, Inc. Dallas, Texas, USA	1:50
**Anti-GRP75**	Western Blot	ab2799	Abcam Cambridge, MA, USA	1:250
**Anti-GRP78**	Western Blot	ab21685	Abcam Cambridge, MA, USA	1:250
**Anti-DDIT3**	Western Blot	ab11419	Abcam Cambridge, MA, USA	1:250

**Anti-GAPDH**	Western Blot (loading control)	ab8245	Abcam Cambridge, MA, USA	1:25000
**Anti-β-actin**	Western Blot (loading control)	HRP-60008	ProteinTech Group, Inc. Rosemont, IL, USA	1:5000
**Goat anti-rabbit IgG (H+L)-HRP-Conjugate**	Western Blot (secondary antibody)	170-6515	BioRad Hercules, CA, USA	1:3000
**Goat anti-mouse IgG (H+L)-HRP-Conjugate**	Western Blot (secondary antibody)	170-6516	BioRad Hercules, CA, USA	1:3000

Supplementary Table S2. List of primers and probes

Gene Sequenced	Primer Direction	Primer/Probe Sequence	Amplicon Size	Primer Bank ID
**ACADVL**	Forward	5'-ACAGATCAGGTGTTCCCATACC-3'	114bp	76496473c1
	Reverse	5'-CTTGGCGGGATCGTTCACTT-3'		
**ACADM**	Forward	5'-TGGATAACCAACGGAGGAAAAG-3'	118bp	187960096c2
	Reverse	5'-CTGGGGTATCTGCTTCCACA-3'		
**IVD**	Forward	5'-ATGGCAGAGATGGCGACTG-3'	135bp	226958411c1
	Reverse	5'-TAGCCCATTGATTGCATCGTC-3'		
**ETFDH**	Forward	5'-TACTGTGCCTCGAATTACTACCC-3'	165bp	119703745c1
	Reverse	5'-ACAGCCAACTGTTTTAGACGAA-3'		
**UQCRC2**	Forward	5'-TTCAGCAATTTAGGAACCACCC-3'	120bp	50592987c1
	Reverse	5'-GGTCACACTTAATTTGCCACCAA-3'		
**TOMM20**	Forward	5'-GGTACTGCATCTACTTCGACCG-3'	220bp	208609996c1
	Reverse	5'-TGGTCTACGCCCTTCTCATATTC-3'		
**GAPDH**	Forward	5'-ACAACTTTGGTATCGTGGAAGG-3'	101bp	378404907c2
	Reverse	5'-GCCATCACGCCACAGTTTC-3'		
**Hs_mtND1**	Forward	5'-CCCTAAAACCCGCCACATCT-3'	125bp	-
	Reverse	5'-GCCTAGGTTGAGGTTGACCA-3'		
	HEX Probe	5'-ACCATCACCCTCTACATCACCGC-3'	-	
**Hs_B2M_in1**	Forward	5'-GGAGAGCTGTGGACTTCGTC-3'	110bp	-
	Reverse	5'-TTCTACAAACGTCGCGTGCTG-3'		
	FAM Probe	5'-CCGCTAAGTTCGCATGTCCTAGCA-3'	-	
**Hs_mtCYB**	Forward	5'-TTCTCCGATCCGTCCCTAACA-3'	132bp	-
	Reverse	5'-GTGATTGGCTTAGTGGGCGAA-3'		
	HEX Probe	5'-AGGCGTCCTTGCCCTATTACTATC-3'	-	
**Hs_RPP30_in 1**	Forward	5'- TTCCTAGCGCGGGAAACTCG-3'	101bp	-
	Reverse	5'- TGCAAATCCCTCGCCCTCGT-3'		
	FAM Probe	5'- TCCTGCAATGAGGGAACTGAGGC-3'	-	

Supplementary Table S3. List of Kits and Reagents Used

Kit	Catalog Number	Company
PCR Mycoplasma Detection Kit	#G238	Applied Biological Materials Richmond, Canada
DC™ Protein Assay Kit	#5000209	Bio-Rad Laboratories Hercules, CA, USA
Seahorse XF Cell Mito Stress Test Kit	#103015-100	Agilent Santa Clara, CA, USA
ATPlite™ Kit	#6016941	PerkinElmer Inc. Waltham, MA, USA
Seahorse XF Real-Time ATP Rate Assay Kit	#103592-100	Agilent Santa Clara, CA, USA
MitoSox Red Dye	#M36008	Invitrogen Grand Island, NY, USA
Mitotracker Green Dye	#M7514	Invitrogen Grand Island, NY, USA
JC-1 Dye	#T3168	Invitrogen Grand Island, NY, USA
100 uM tritiated [9,10-^{3}H]oleate	#NET289001MC	PerkinElmer Inc. Waltham, MA, USA
RNeasy Mini Kit	#74104	Qiagen Valencia, CA, USA
SuperScript™ IV VILO™ Master Mix	#11756050	Invitrogen Grand Island, NY, USA
PowerUp™ SYBR™ Green Master Mix	#A25742	ThermoFisher Scientific Waltham, MA, USA
Hoescht Blue Stain	#H3570	Invitrogen Grand Island, NY, USA

Supplementary Figure S1. Full uncropped western blots for **Figure 1a**, using anti-C22orf25, antibody on whole cell extract from fibroblasts from patient and control cell lines. Anti-GAPDH antibody was used as a loading control. Protein loaded was 25µg.

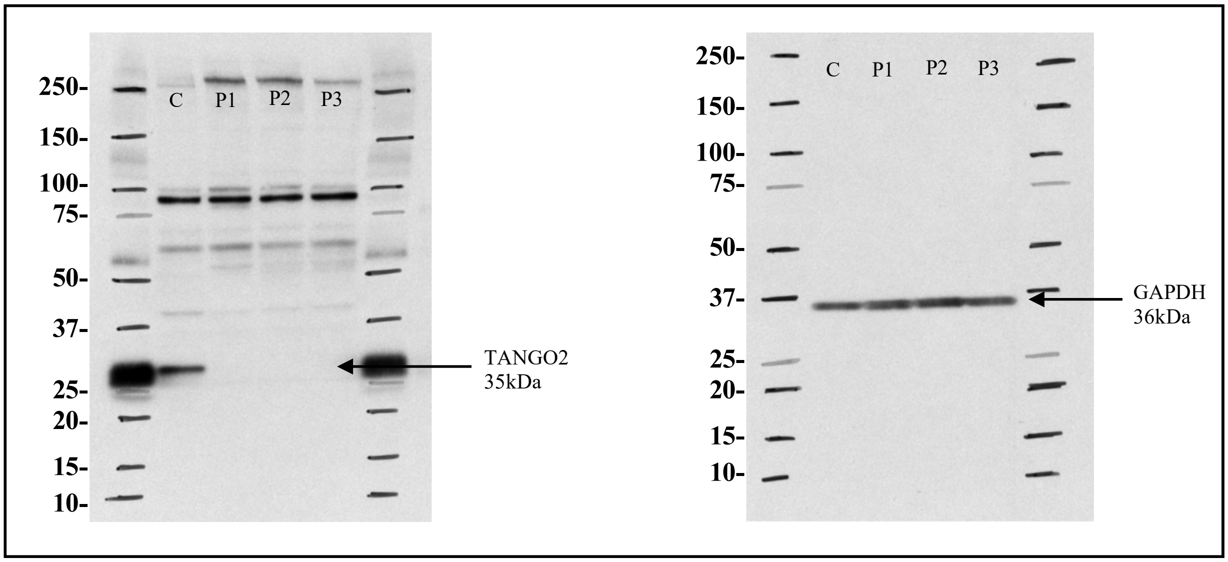

Supplementary Figure S2. Full uncropped western blots for **Figure 1b**, using anti-C22orf25, antibody on mitochondrial extract from fibroblasts from patient and 2 control cell lines. Anti-β-actin antibody and anti-Hsp60 were used as loading controls. Protein loaded was 30μg.

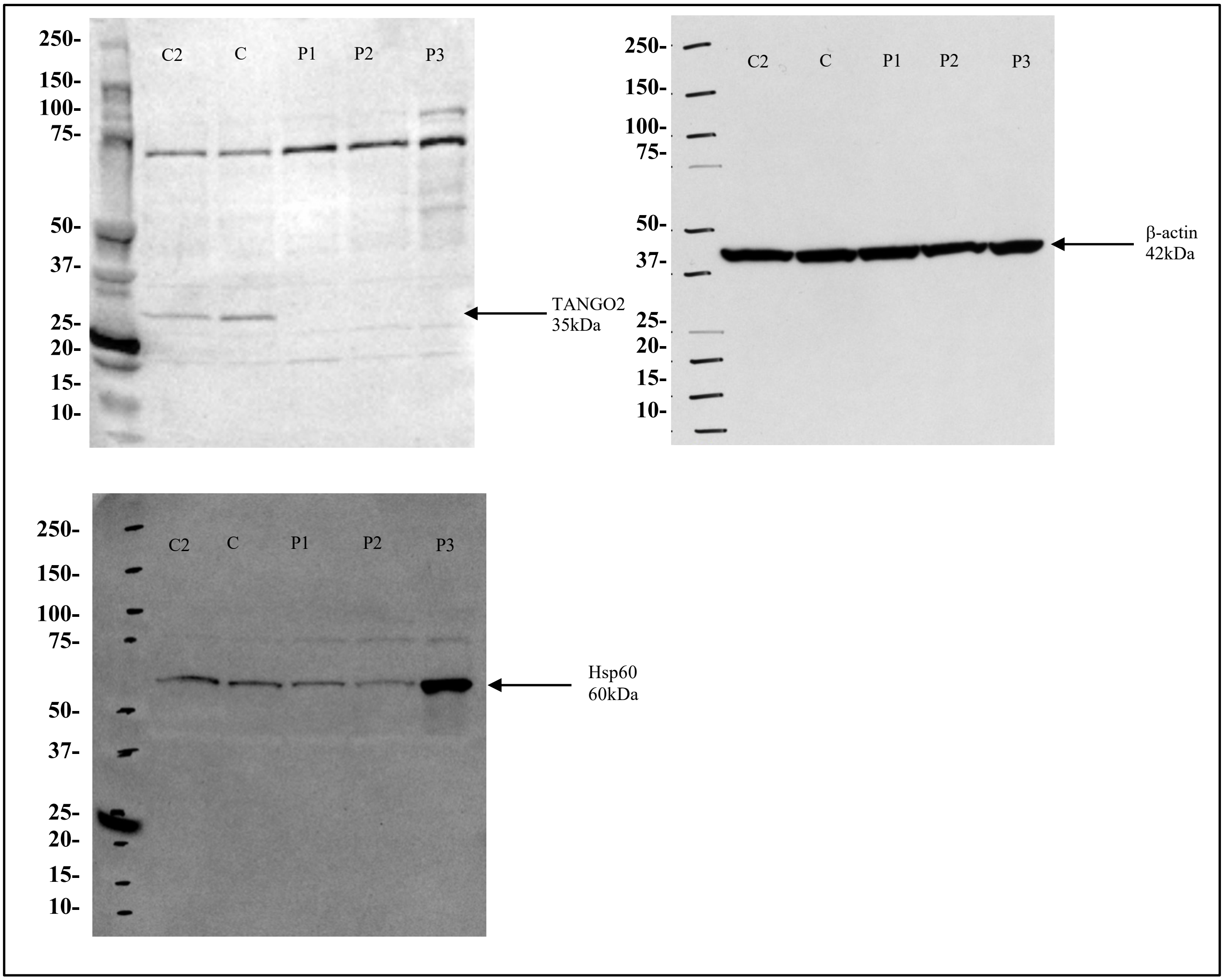

Supplementary Figure S3. Full uncropped western blots for **Figure 3c**, using antibodies for fusion-associated proteins including anti-MFN1, anti-MFN2, and anti-OPA1 on whole cell extract from fibroblasts from patient and control cell lines. Anti-GAPDH antibody was used as a loading control. Protein loaded was 25μg.

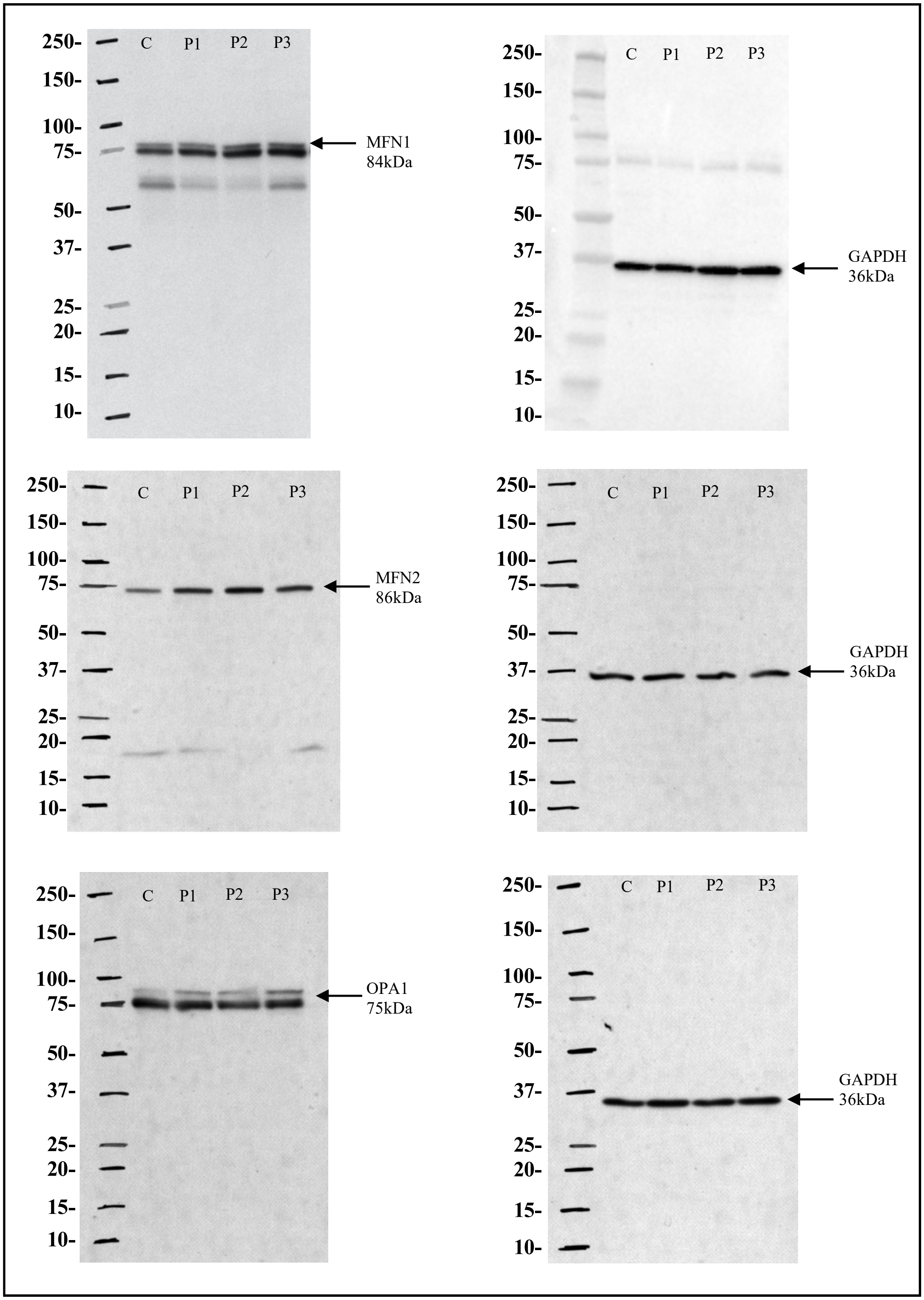

Supplementary Figure S4. Full uncropped western blots for **Figure 3c**, using antibody for fission-associated protein anti-DRP1 on whole cell extract from fibroblasts from patient and control cell lines. Anti-GAPDH antibody was used as a loading control. Protein loaded was 25μg.

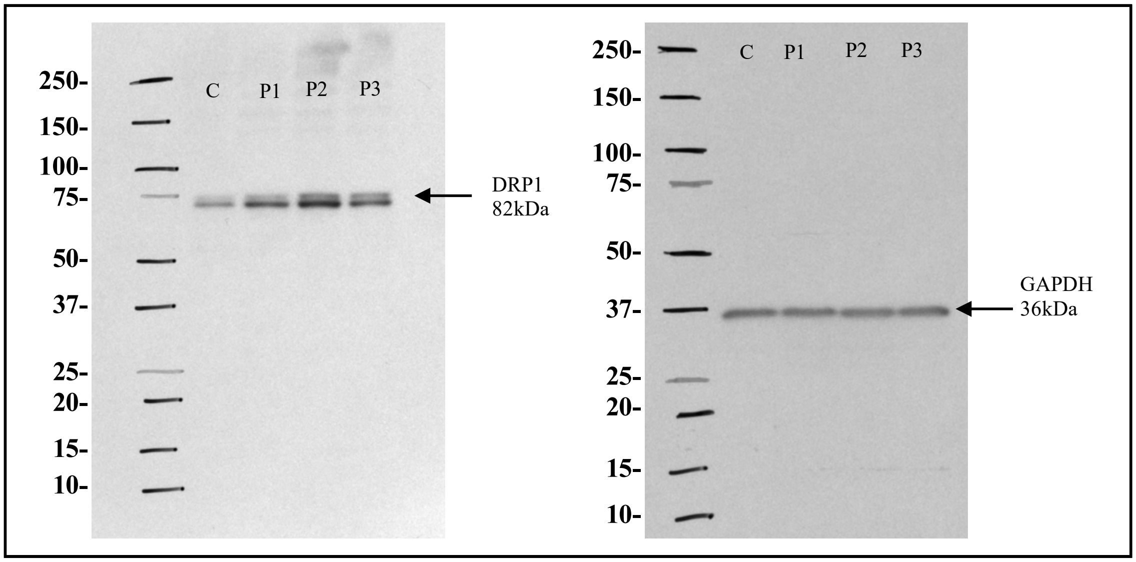

Supplementary Figure S5. Full uncropped western blots for **Figure 3c**, using antibodies for proteins associated with ER-Mitochondrial crosstalk including anti-GRP75 and anti-IP3R on whole cell extract from fibroblasts from patient and control cell lines. Anti-GAPDH antibody was used as a loading control. Protein loaded was 25µg.

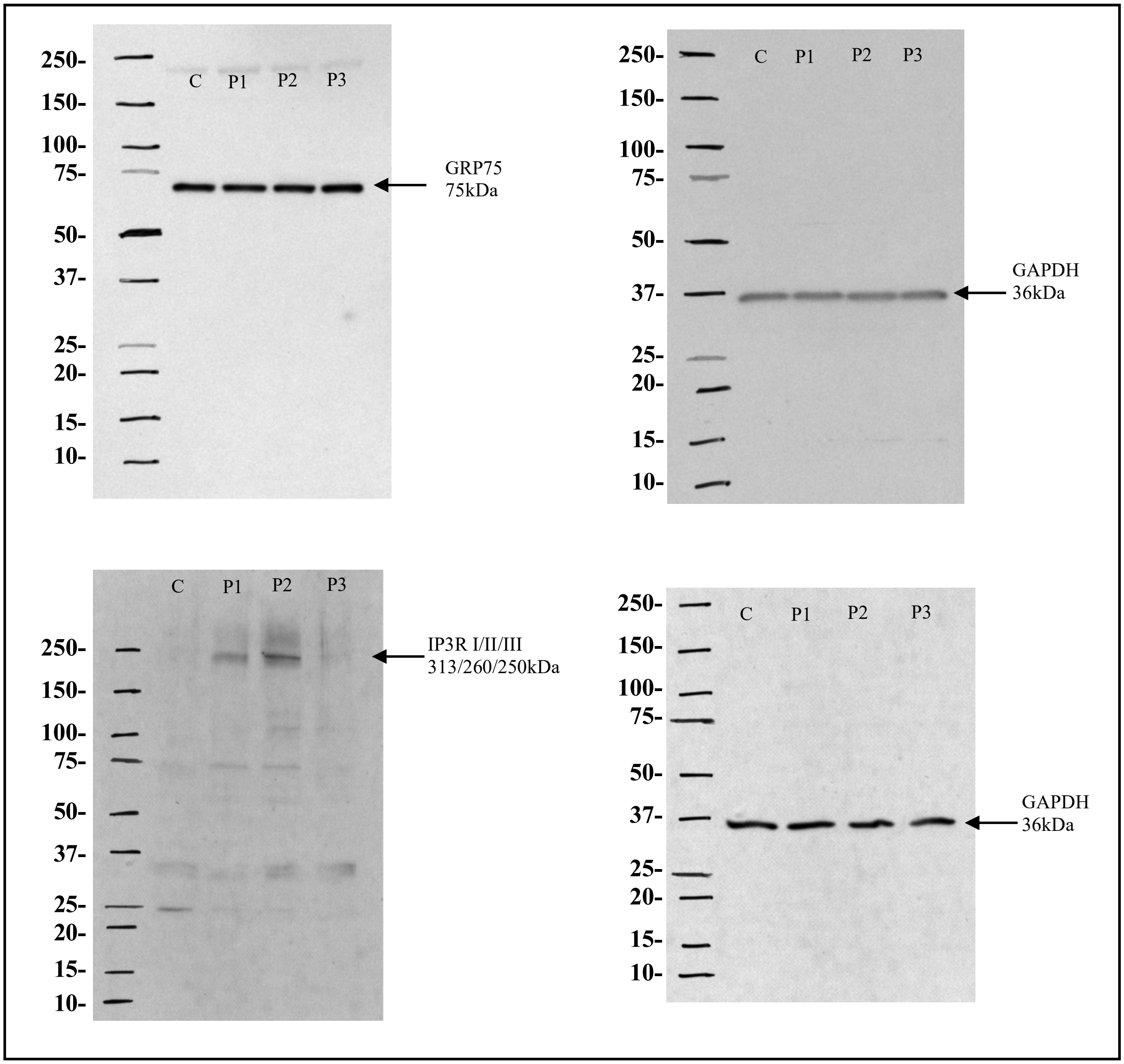

Supplementary Figure S6. Full uncropped western blots for **Figure 3c**, using antibodies for proteins associated with ER Stress including anti-DDIT3 and anti-GRP78 on whole cell extract from fibroblasts from patient and control cell lines. Anti-GAPDH antibody was used as a loading control. Protein loaded was 25μg.

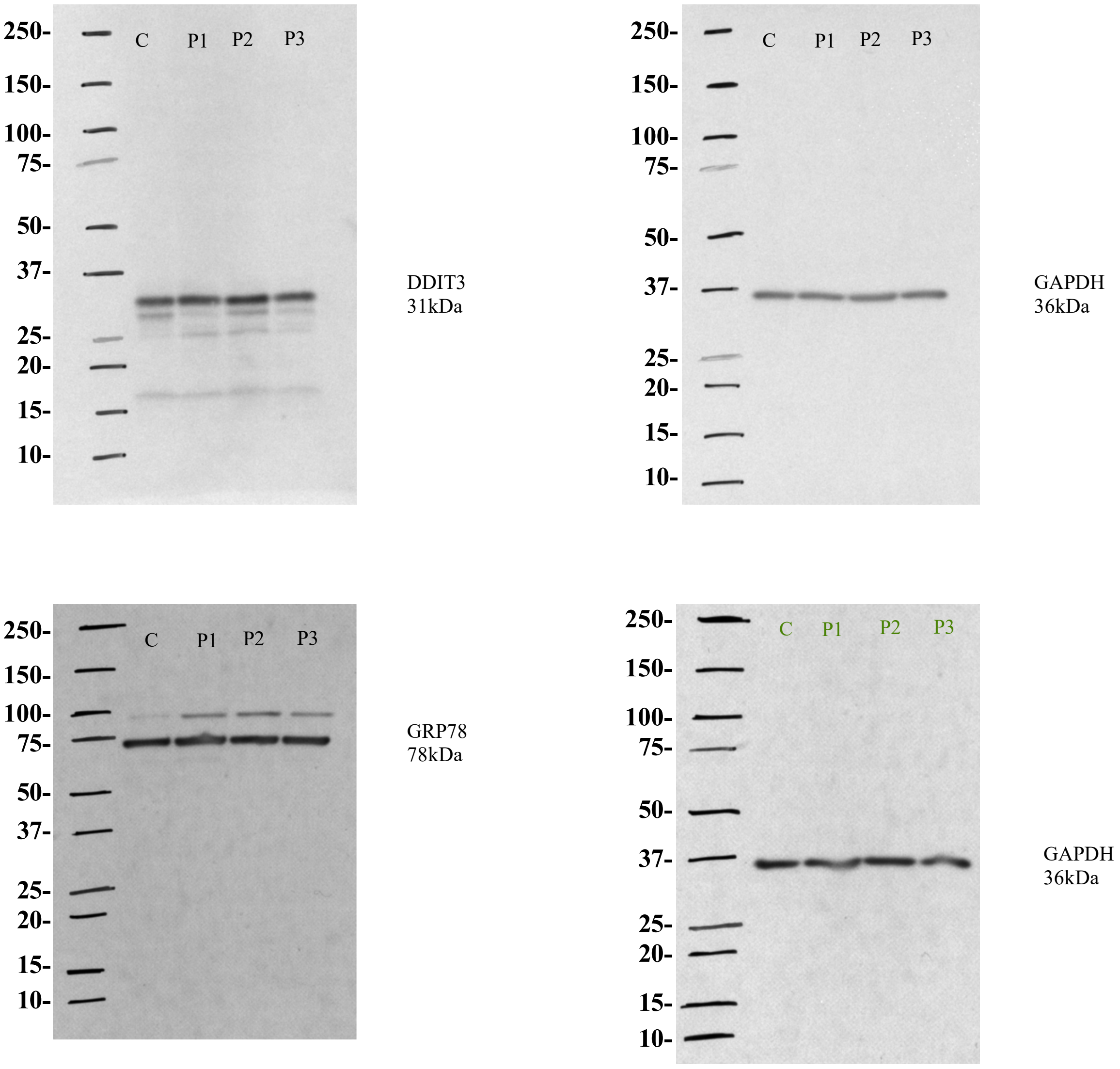

Supplementary Figure S7. Full uncropped western blots for **Figure 4b**, using anti-VLCAD, anti-MCAD, and anti-ETFDH, antibodies on whole cell extracts from fibroblasts from patient and control cell lines. Anti-GAPDH and anti-AK2 antibodies were used as cytosolic and mitochondrial loading controls, respectively. Protein loaded was 25μg.

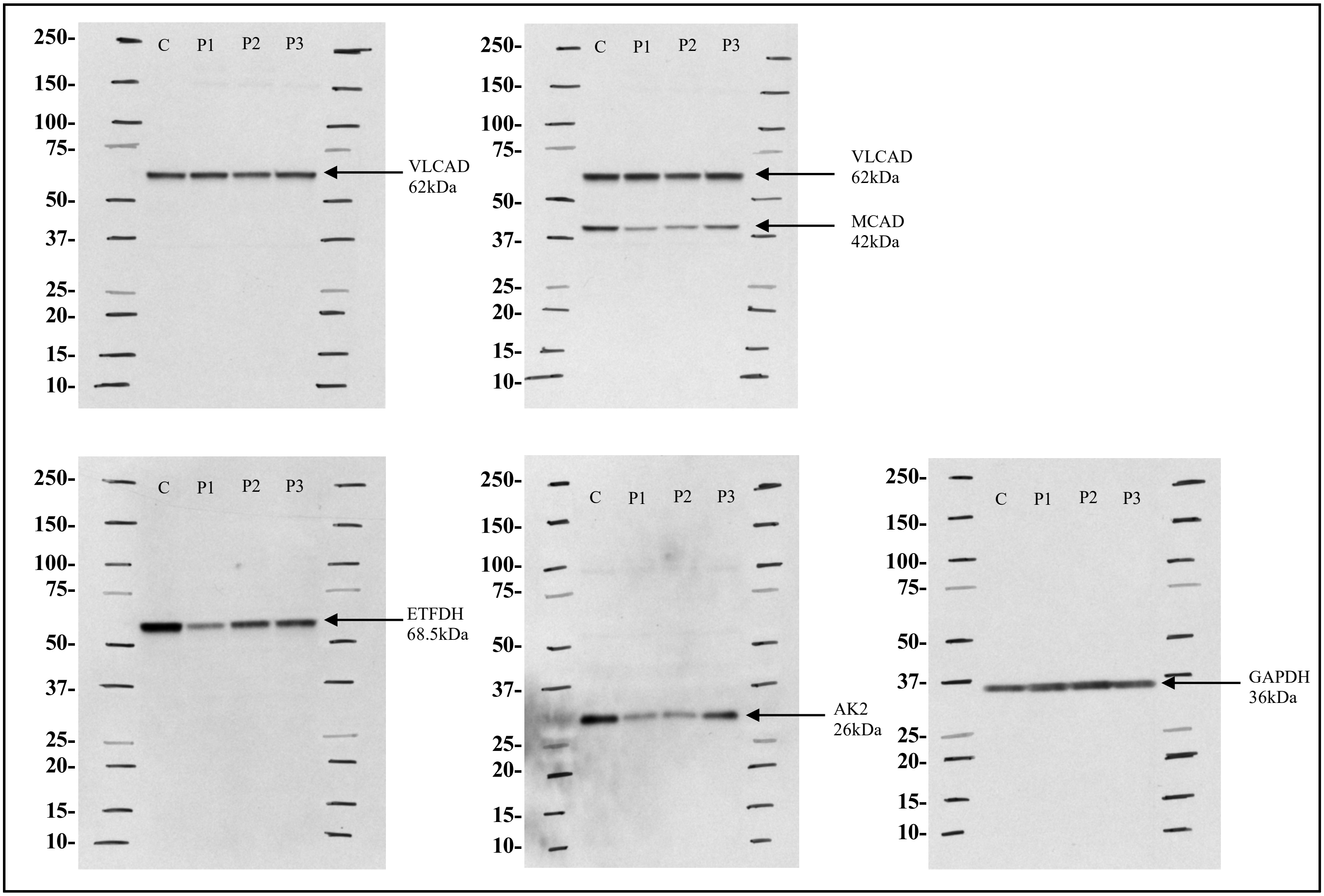

Supplementary Figure S8. Full uncropped western blots for **Figure 4b**, using anti-IVD, anti-Hsp60, and anti-TOMM20 antibodies on whole cell extracts from fibroblasts from patient and control cell lines. Anti-GAPDH and anti-AK2 antibodies were used as cytosolic and mitochondrial loading controls, respectively. Protein loaded was 25µg.

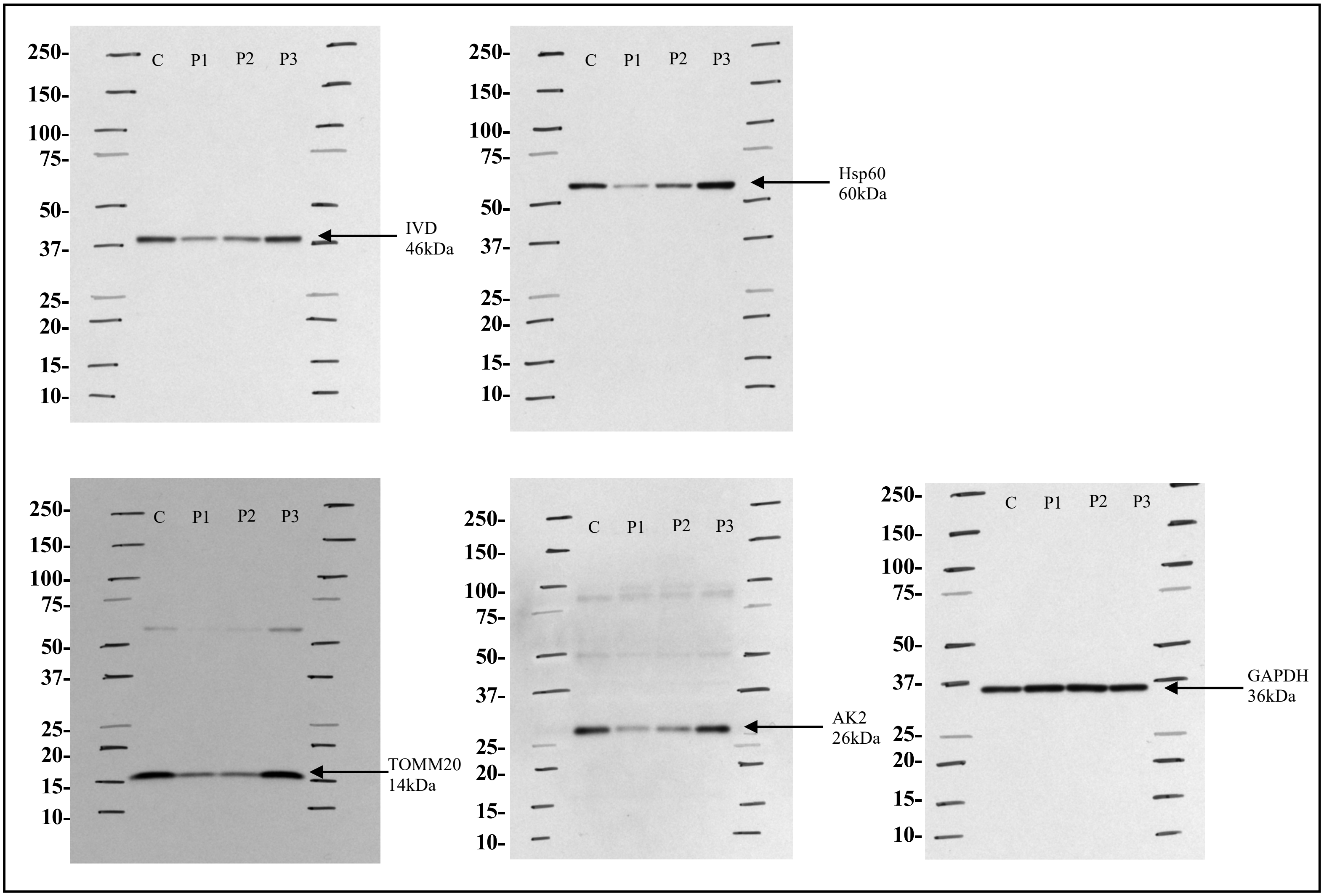

Supplementary Figure S9. Full uncropped western blots for **Figure 4b**, using an OXPHOS antibody cocktail and anti-MTCO1 antibody on whole cell extracts from fibroblasts from patient and control cell lines. Anti-GAPDH and anti-AK2 antibodies were used as cytosolic and mitochondrial loading controls, respectively. Protein loaded was 25μg.

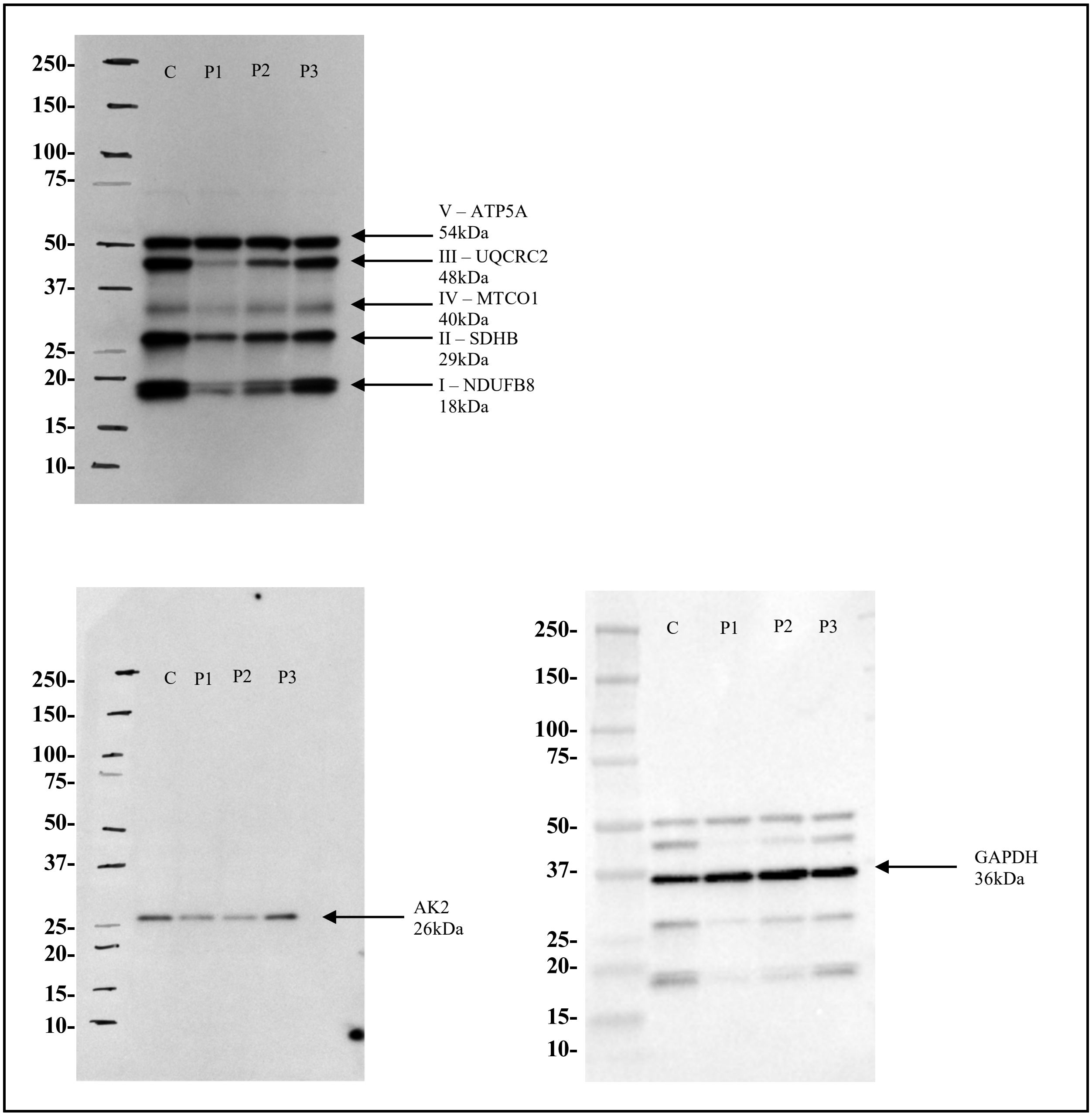

Supplementary Figure S10. Full Statistics for Seahorse ATPrate Assay

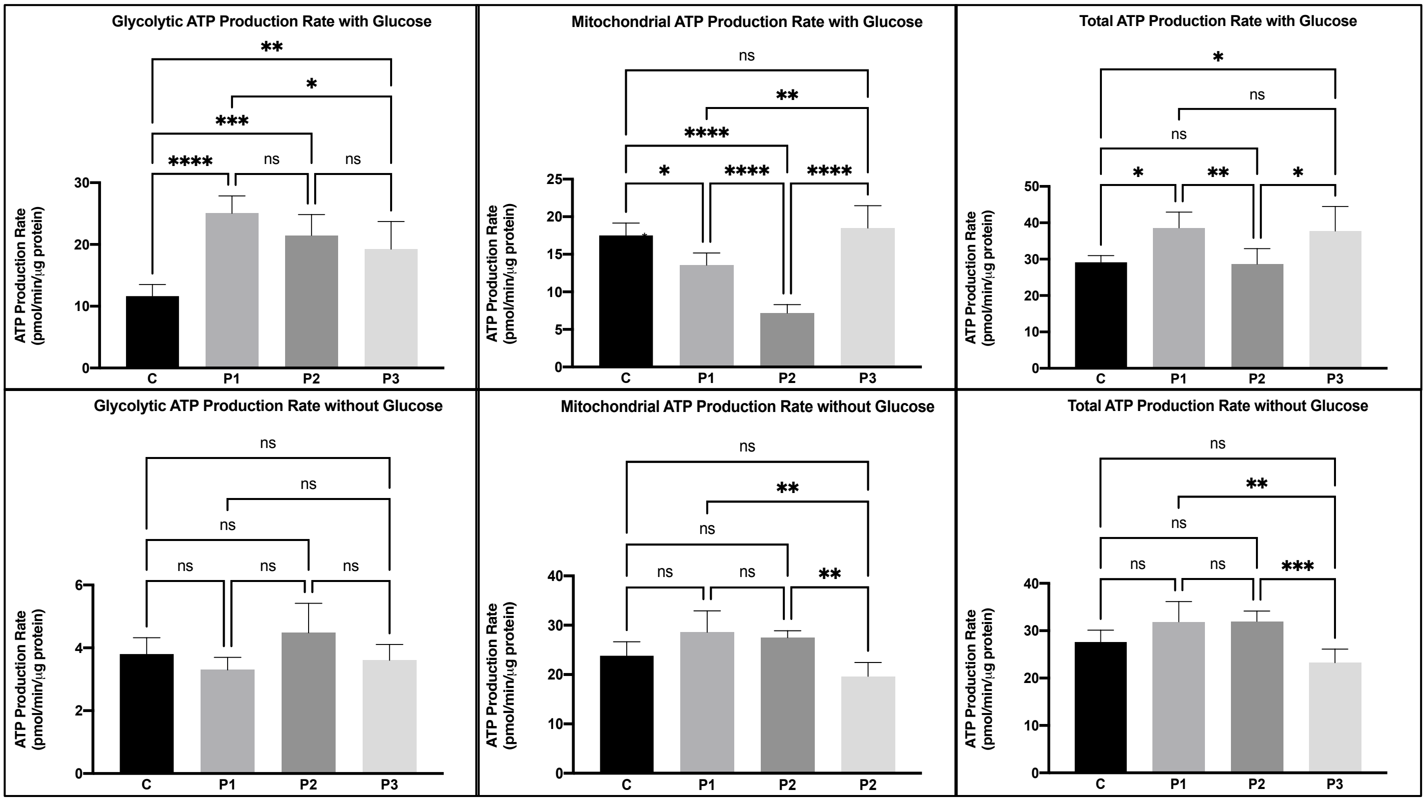

Supplementary Figure S11. Droplet digital PCR 1D amplitude heat-map graphs from mtDNA copy number assays. (**A**) B2M/mtND1 assay (**B**) RPP30/mtCYB assay. Blue dots above baseline represent positive droplets. Concentration calculated by Poisson distribution noted above each technical triplicate reaction. Concentration of diploid nuclear genomic reference calculated based on undiluted DNA whereas the concentration of mtDNA was based on 1:100 diluted sample.

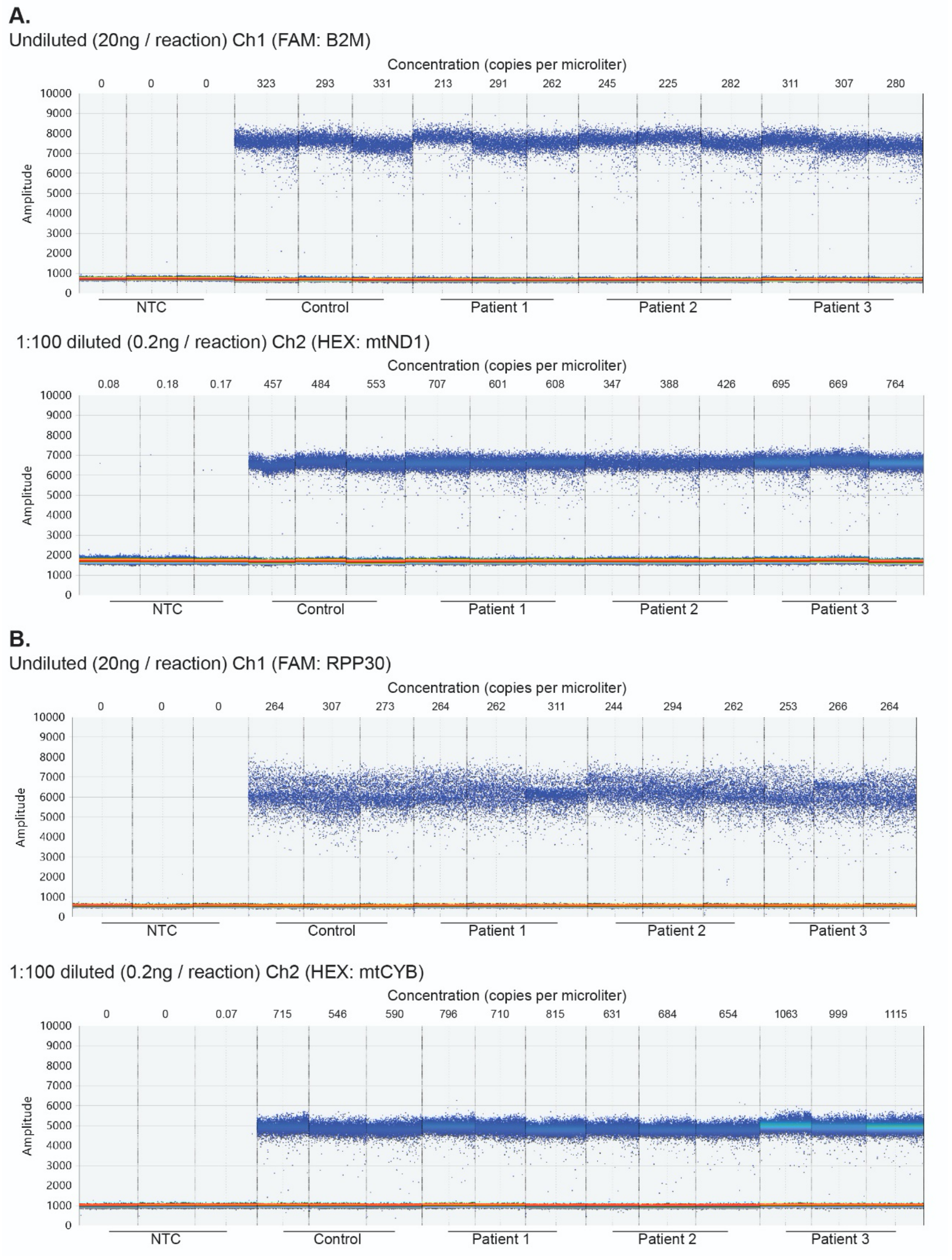

Supplement: Supplementary file 1 — Supplementary Information 1. [file 41598_2022_7076_MOESM1_ESM.pdf]
