# Supplementary material for: Mitochondrial dysfunction associated with TANGO2 deficiency
Source: Sci Rep. 2022 Feb 23;12:3045. doi: 10.1038/s41598-022-07076-9 (PMC8866466; doi:10.1038/s41598-022-07076-9)

**Supplementary Figure S1.** Full uncropped western blots for **Figure 1a**, using anti-C22orf25, antibody on whole cell extract from fibroblasts from patient and control cell lines. Anti-GAPDH antibody was used as a loading control. Protein loaded was 25μg.

Figure S1: Anti-C22orf25 (anti-TANGO2) whole cell western blot (left side)


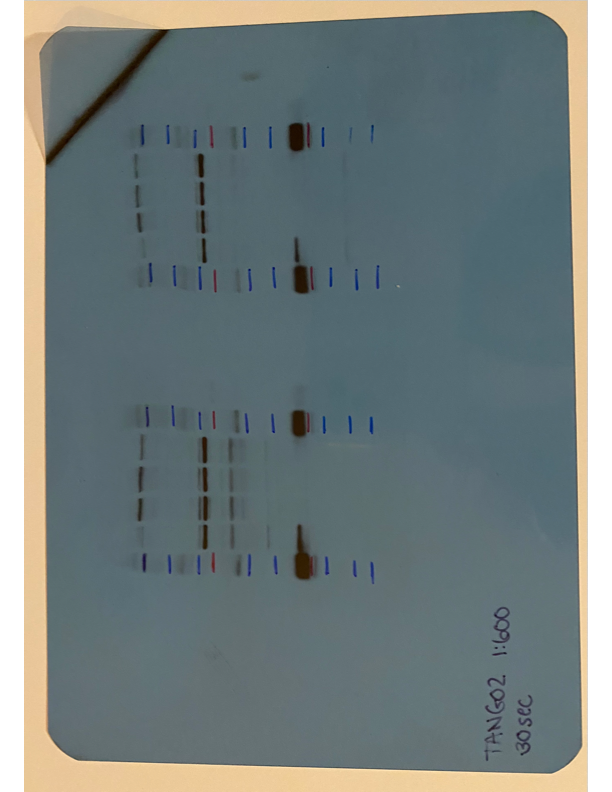


Figure S1: Anti-GAPDH whole cell western blot (left side)


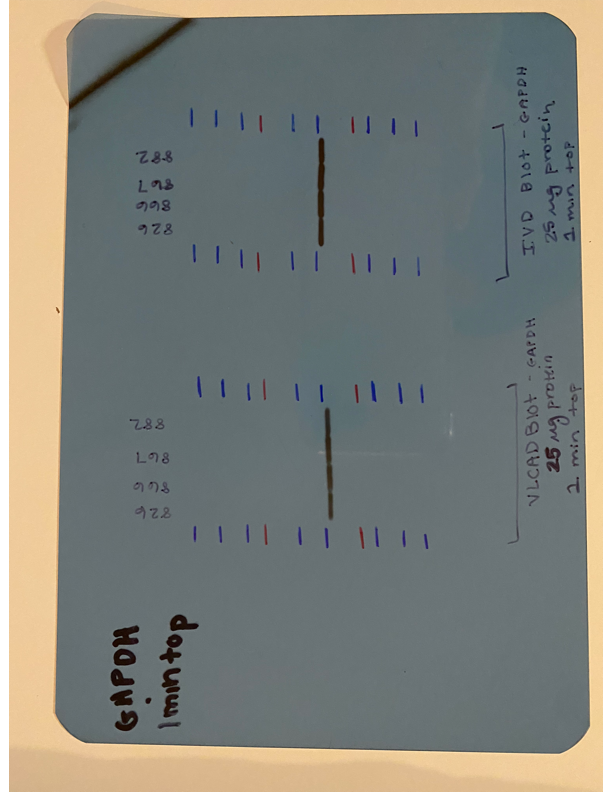


**Supplementary Figure S2.** Full uncropped western blots for **Figure 1b**, using anti-C22orf25, antibody on mitochondrial extract from fibroblasts from patient and 2 control cell lines. Anti-β-actin antibody and anti-Hsp60 were used as loading controls was used as a loading control. Protein loaded was 30μg.

Figure S2: Anti-C22orf25 (anti-TANGO2) mitochondrial extract western blot.  This blot was imaged on the BioRad machine (not X-ray). This is the only image saved for the blot.


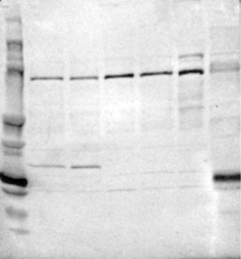


Figure S2: Anti-β-actin antibody mitochondrial extract western blot.


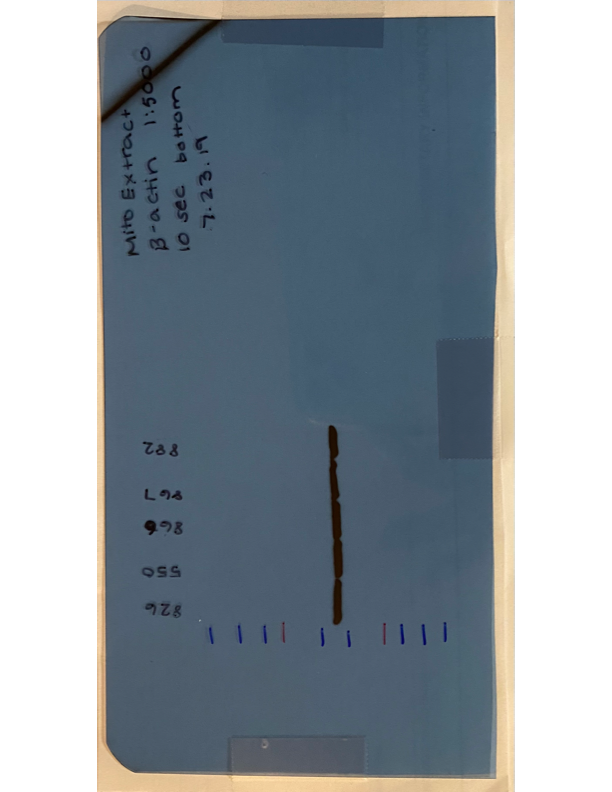


Figure S2: Anti-Hsp60 antibody mitochondrial extract western blot.

**
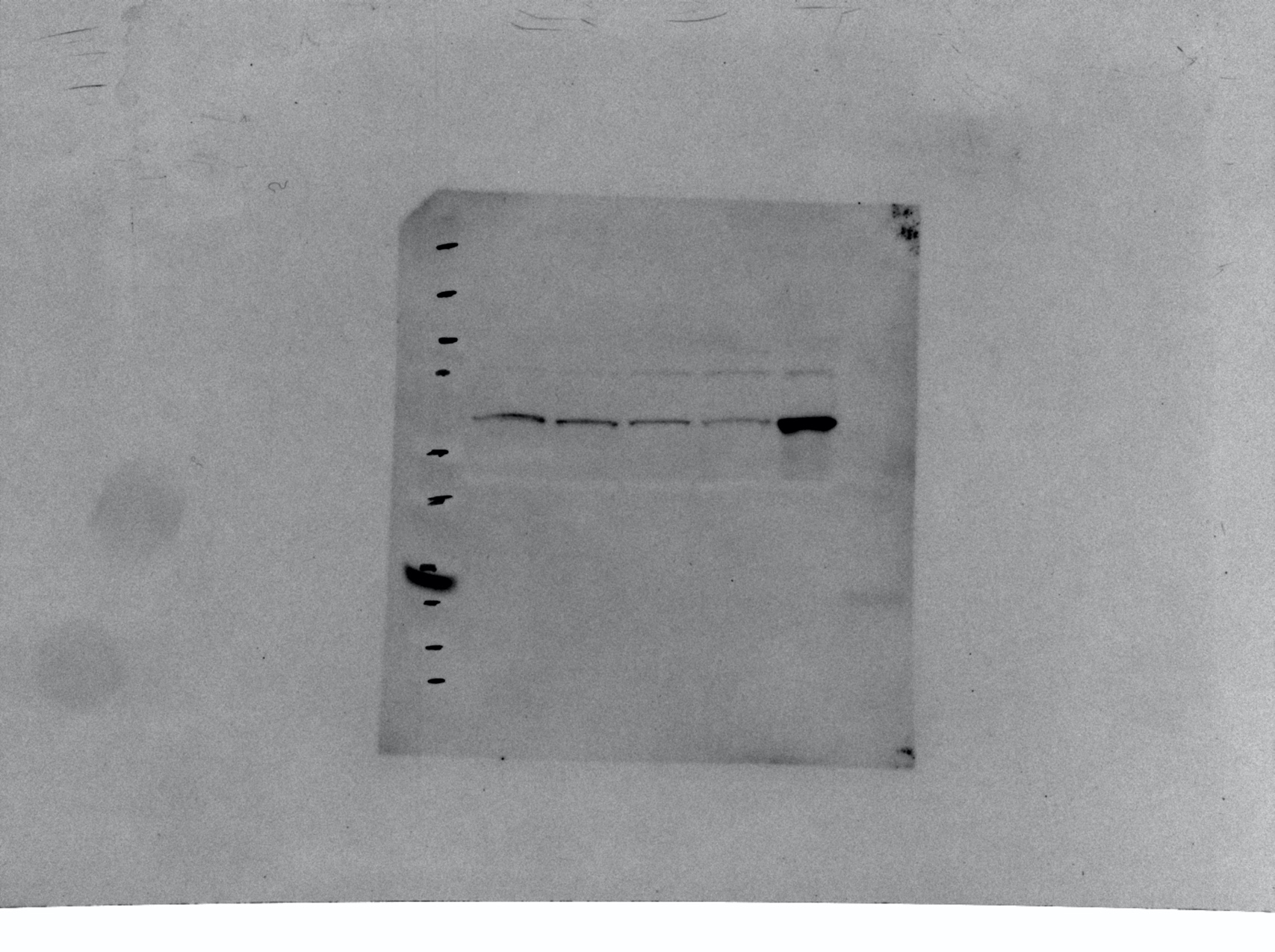
**

**Supplementary Figure S3.** Full uncropped western blots for **Figure 3c**, using antibodies for fusion-associated proteins including anti-MFN1, anti-MFN2, and anti-OPA1 on whole cell extract from fibroblasts from patient and control cell lines. Anti-GAPDH antibody was used as a loading control. Protein loaded was 25μg.

Figure S3: Anti-MFN1 western blot


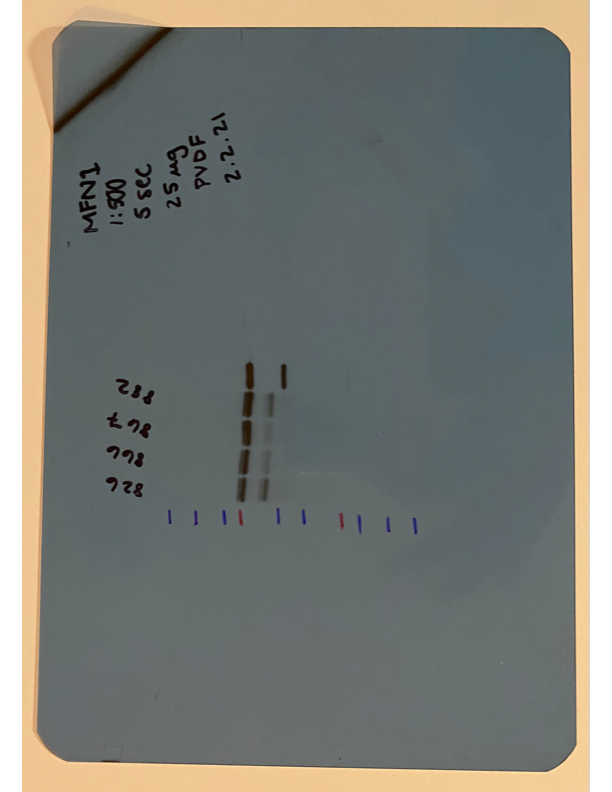


Figure S3: Anti-GAPDH antibody as a loading control for anti-MFN1 antibody. This blot was imaged on the BioRad machine (not X-ray). This is the only image saved for the blot.


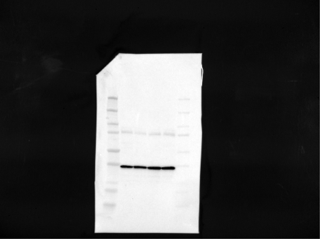


Figure S3: Anti-MFN2 western blot


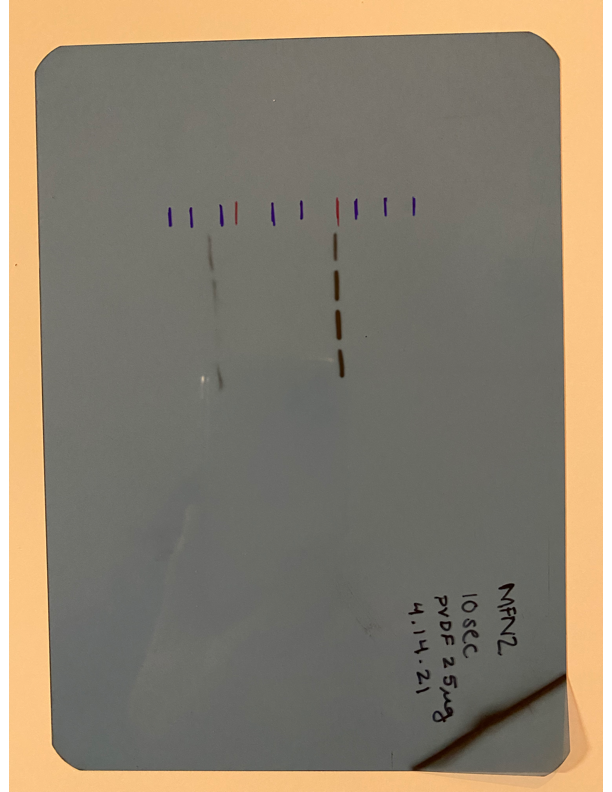


Figure S3: Anti-GAPDH antibody as a loading control for anti-MFN2 antibody.


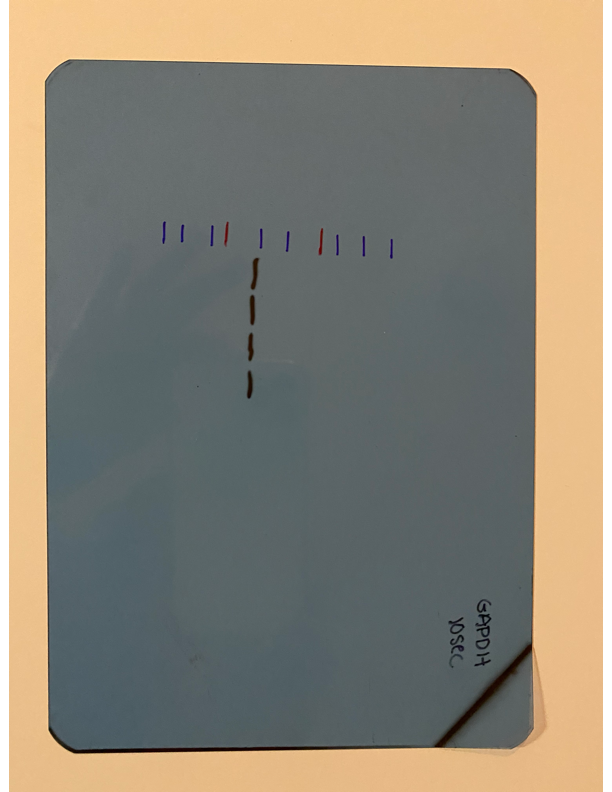


Figure S3: Anti-OPA1 western blot


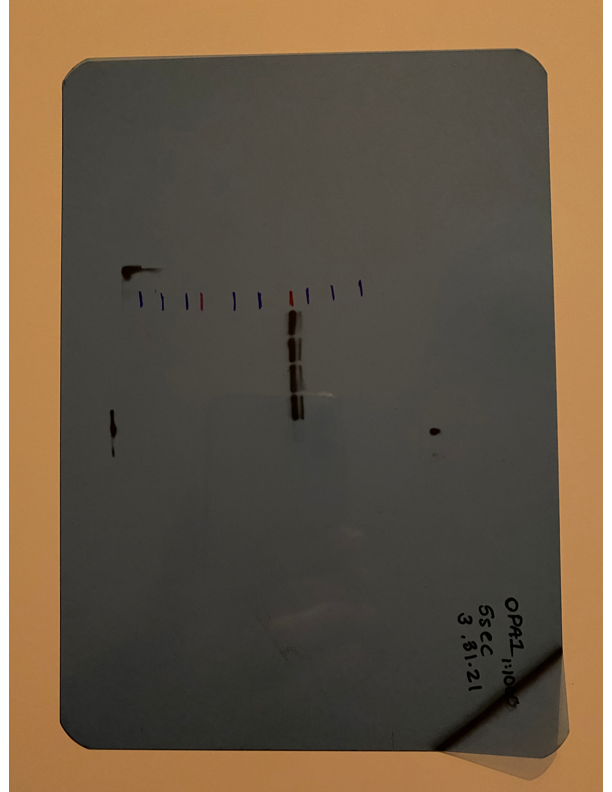


Figure S3: Anti-GAPDH antibody as a loading control for anti-OPA1 antibody.


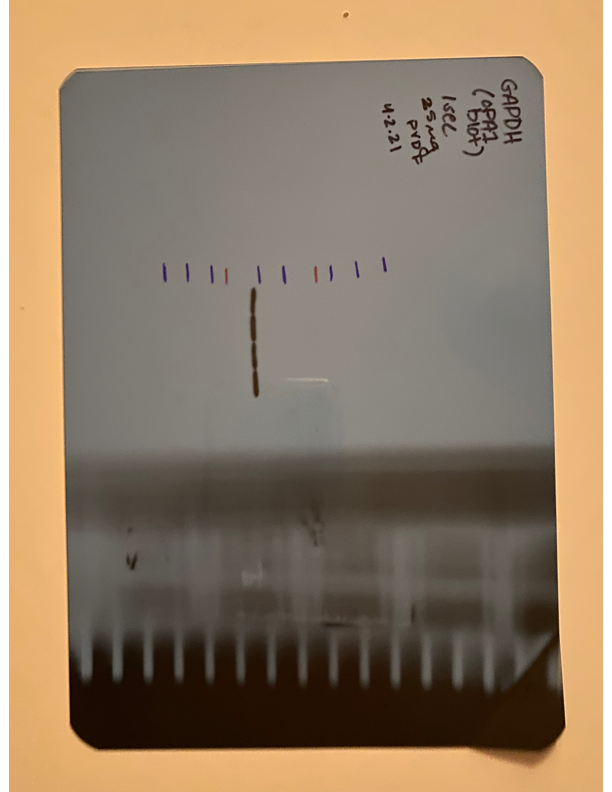


**Supplementary Figure S4.** Full uncropped western blots for **Figure 3c**, using antibody for fission-associated protein anti-DRP1 on whole cell extract from fibroblasts from patient and control cell lines. Anti-GAPDH antibody was used as a loading control. Protein loaded was 25μg.

Figure S4: Anti-DRP1 antibody (right side)


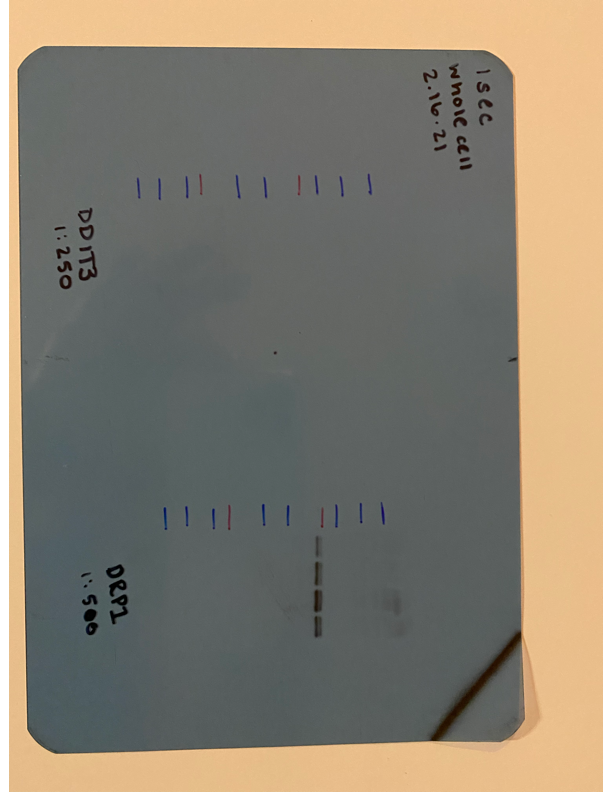


Figure S4: Anti-GAPDH antibody as a loading control for anti-DRP1 antibody.


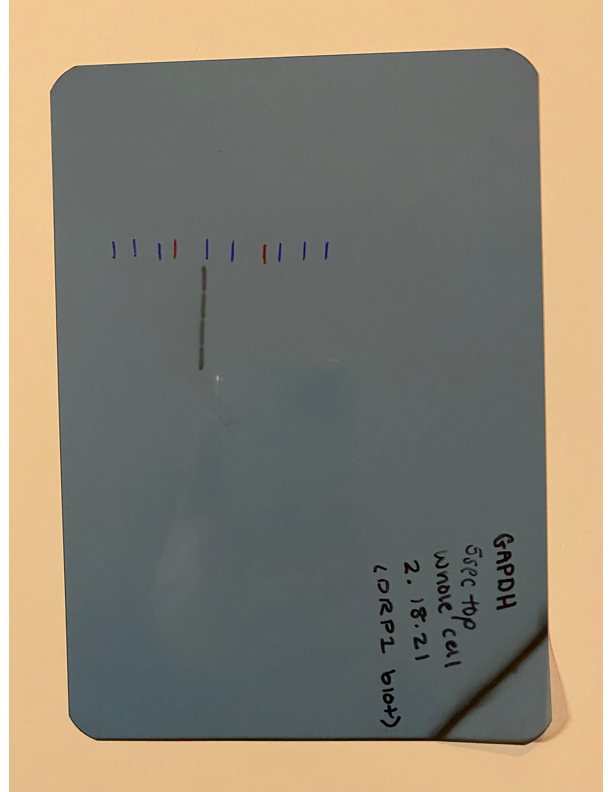


**Supplementary Figure S5.** Full uncropped western blots for **Figure 3c**, using antibodies for proteins associated with ER-Mitochondrial crosstalk including anti-GRP75 and anti-IP3R on whole cell extract from fibroblasts from patient and control cell lines. Anti-GAPDH antibody was used as a loading control. Protein loaded was 25μg.

Figure S5: Anti-GRP75 antibody.


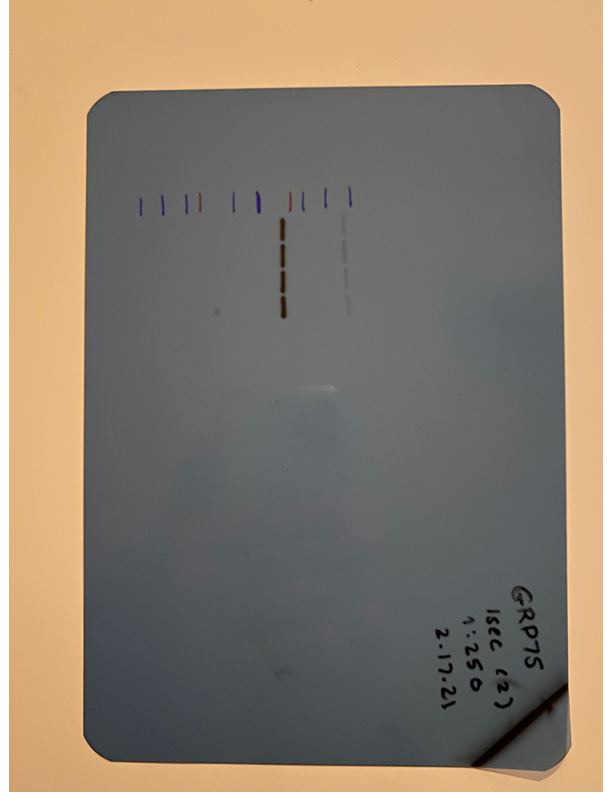


Figure S5: Anti-GAPDH antibody as a loading control for anti-GRP75 antibody (This is the same figure as in Figure S4 as a loading control for anti-DRP1 since this is the same blot).


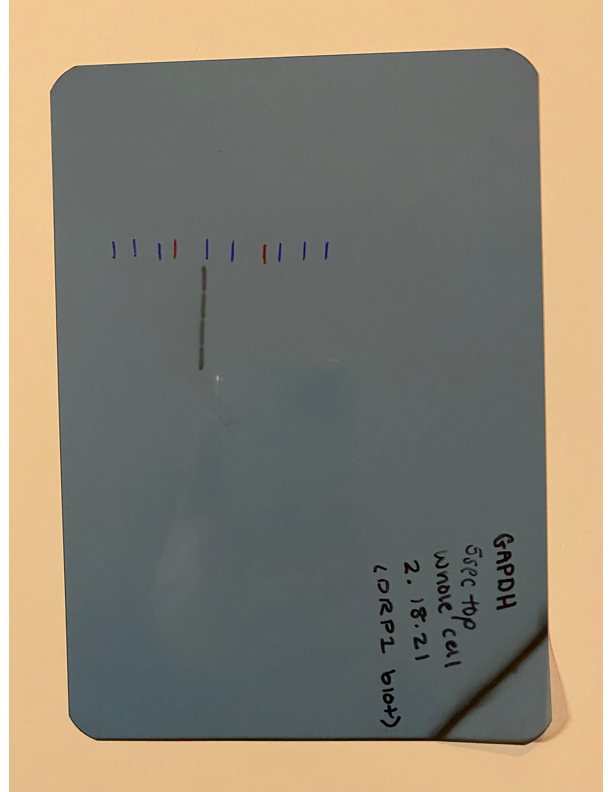


Figure S5: Anti-IP3R antibody.


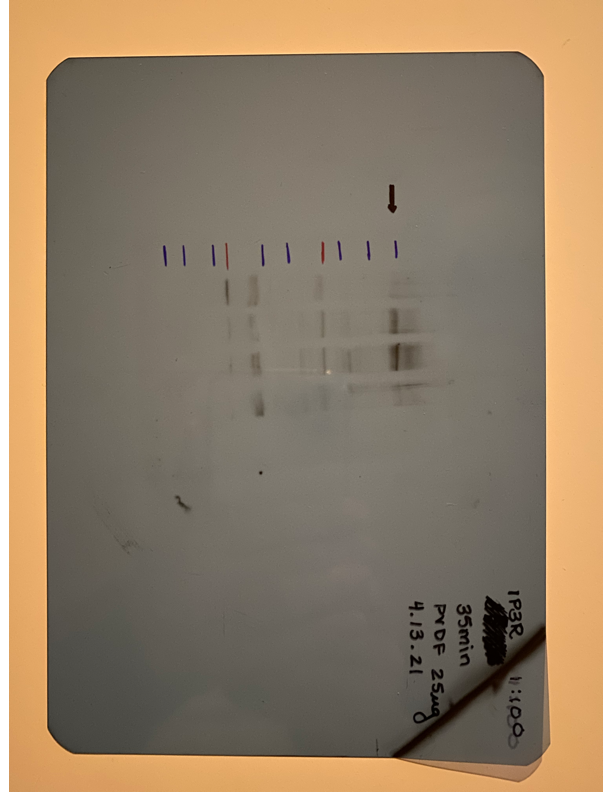


Figure S5: Anti-GAPDH antibody as a loading control for anti-IP3R antibody

**Supplementary Figure S6.** Full uncropped western blots for **Figure 3c**, using antibodies for proteins associated with ER Stress including anti-DDIT3 and anti-GRP78 on whole cell extract from fibroblasts from patient and control cell lines. Anti-GAPDH antibody was used as a loading control. Protein loaded was 25μg.

Figure S6: Anti-DDIT3 antibody (left side)


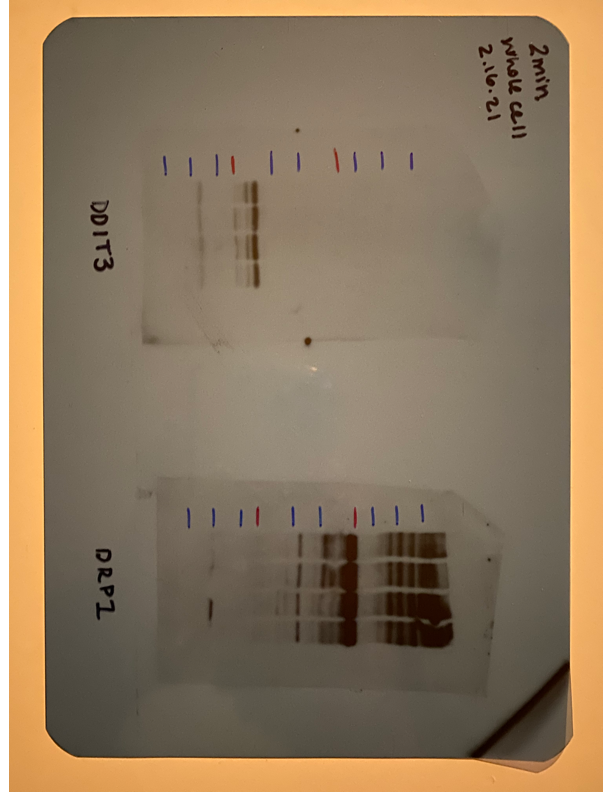


Figure S6: Anti-GAPDH antibody as a loading control for anti-DDIT3 antibody.


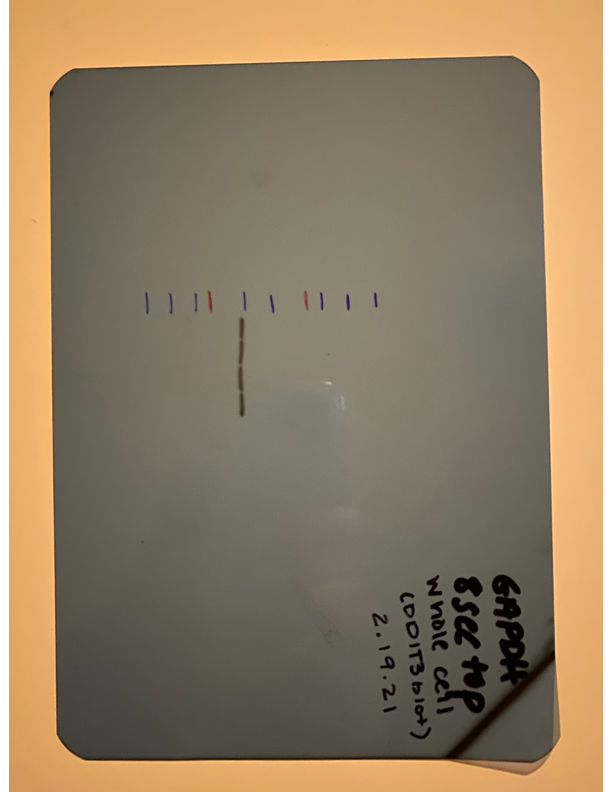


Figure S6: Anti-GRP78 antibody


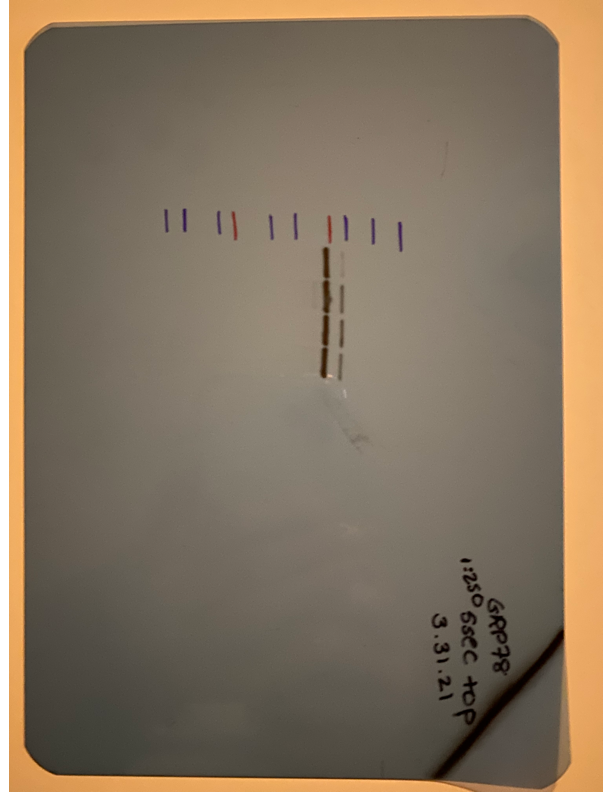


Figure S6: Anti-GAPDH antibody as a loading control for anti-GRP78 antibody

**
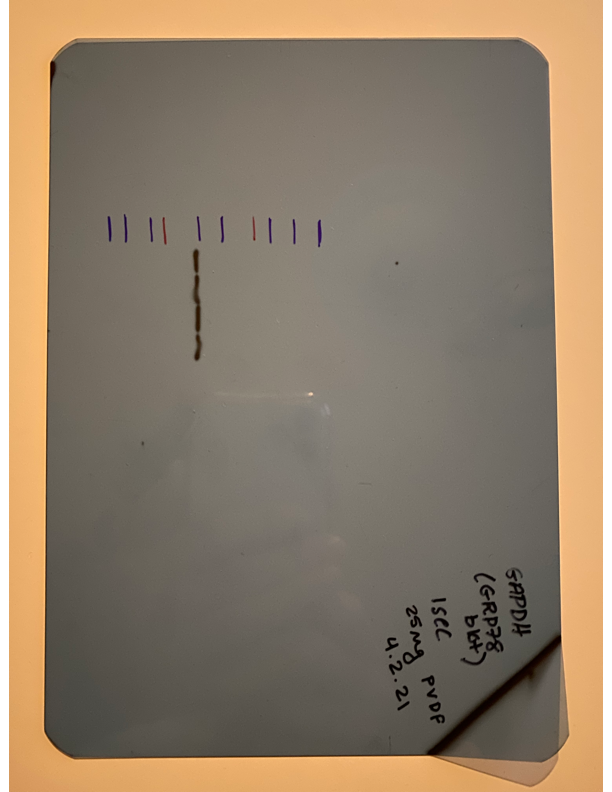
**

**Supplementary Figure S7.** Full uncropped western blots for **Figure 4b**, using anti-VLCAD, anti-MCAD, and anti-ETFDH, antibodies on whole cell extracts from fibroblasts from patient and control cell lines. Anti-GAPDH and anti-AK2 antibodies were used as cytosolic and mitochondrial loading controls, respectively. Protein loaded was 25μg.

Figure S7: Anti-VLCAD antibody (left side)


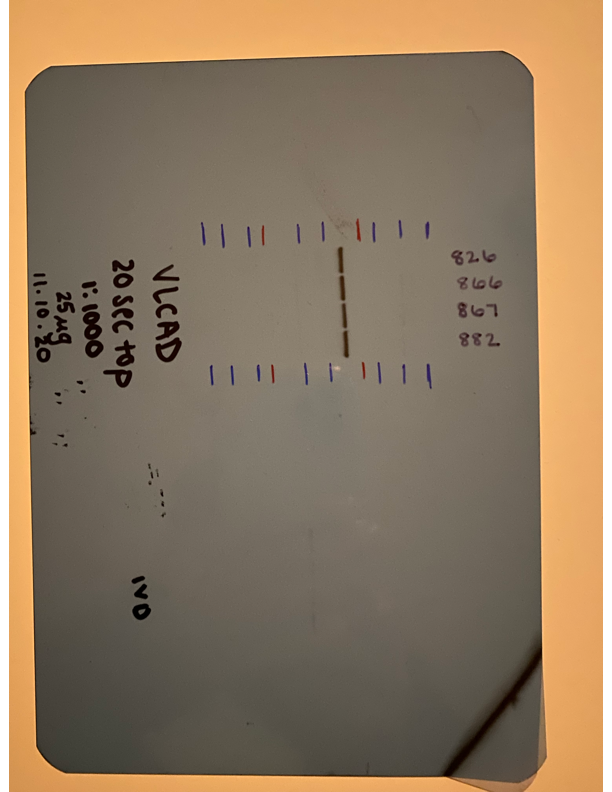


Figure S7: Anti-MCAD antibody


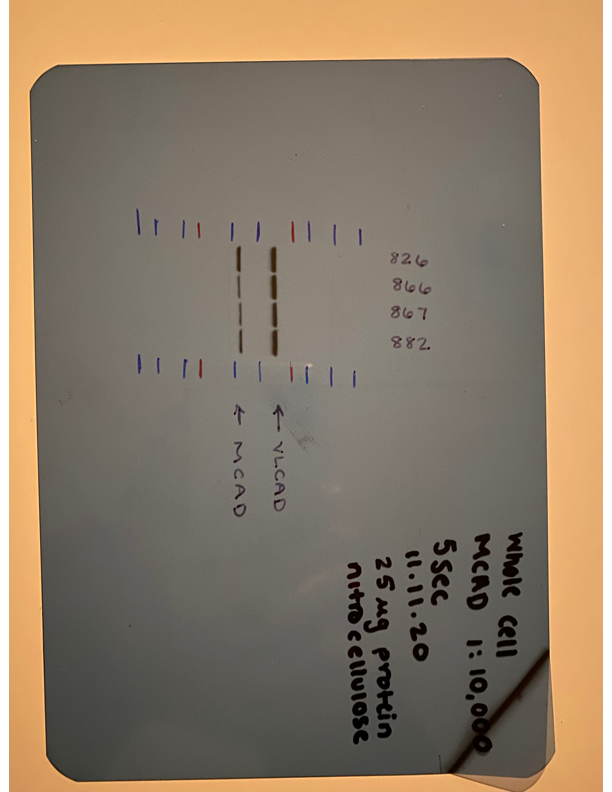


Figure S7: Anti-ETFDH antibody (left side)


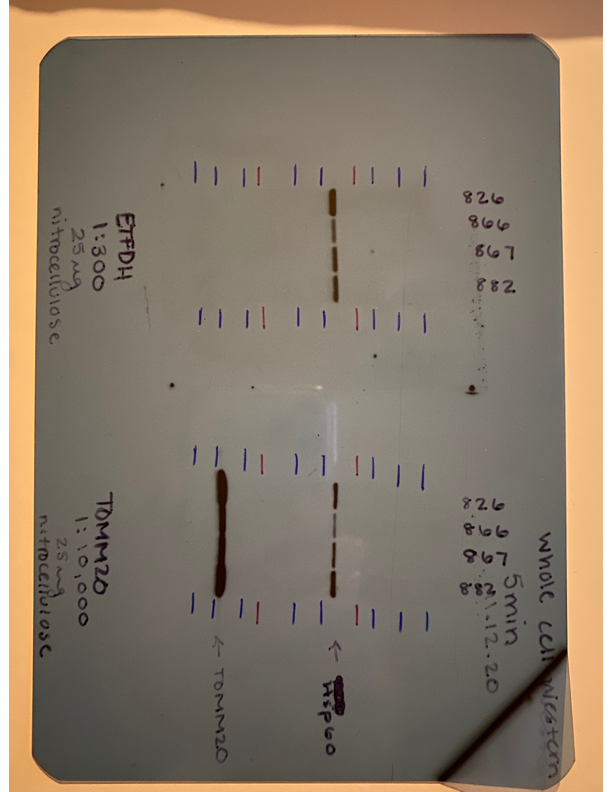


Figure S7: Anti-AK2 antibody


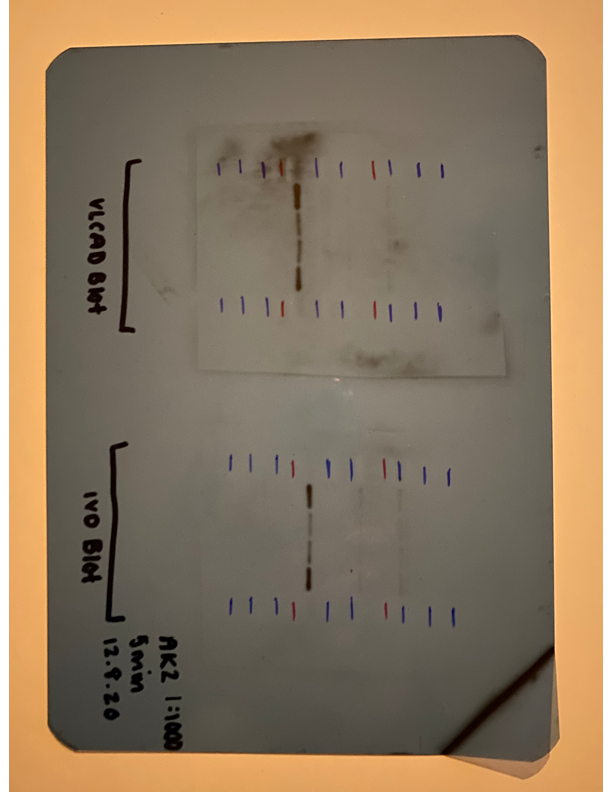


Figure S7: Anti-GAPDH antibody as a loading control

**Supplementary Figure S8.** Full uncropped western blots for **Figure 4b**, using anti-IVD, anti-Hsp60, and anti-TOMM20 antibodies on whole cell extracts from fibroblasts from patient and control cell lines. Anti-GAPDH and anti-AK2 antibodies were used as cytosolic and mitochondrial loading controls, respectively. Protein loaded was 25μg.

Figure S8: Anti-IVD antibody (right side)


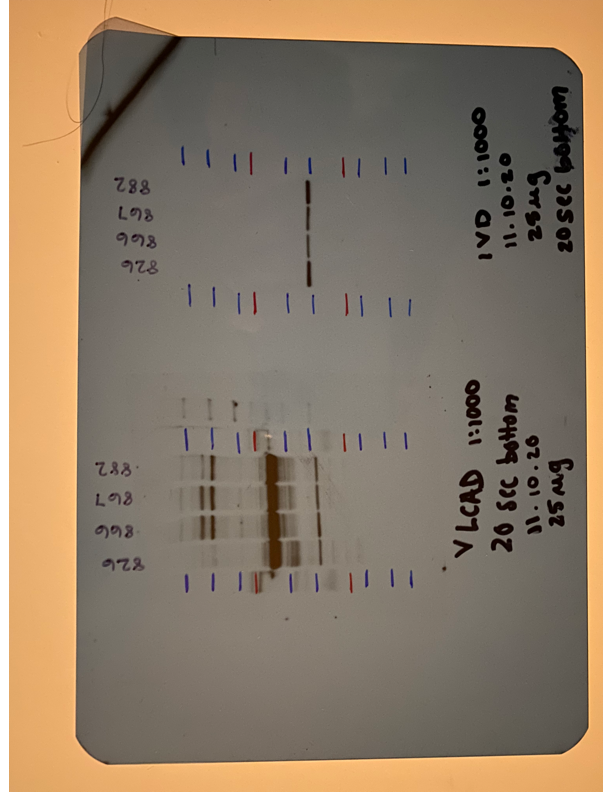


Figure S8: Anti-Hsp60 antibody (left side)


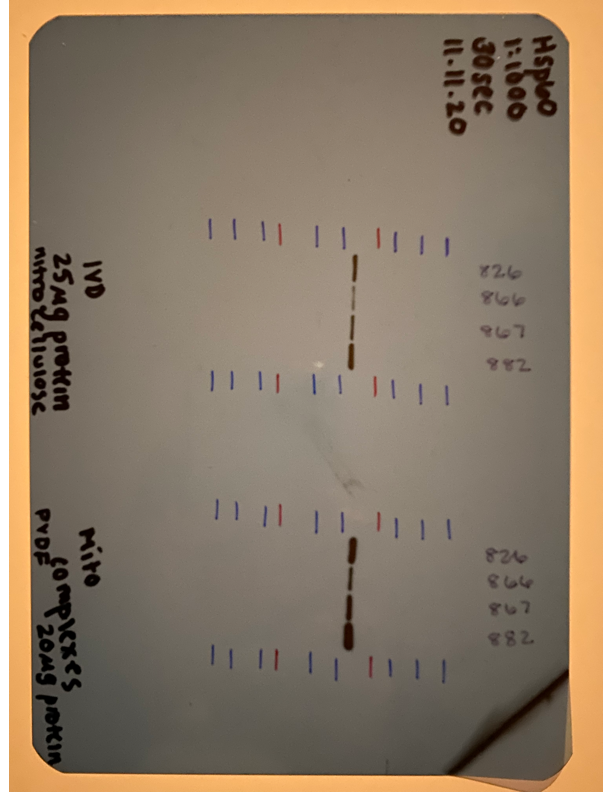


Figure S8: Anti-TOMM20 antibody (right side)


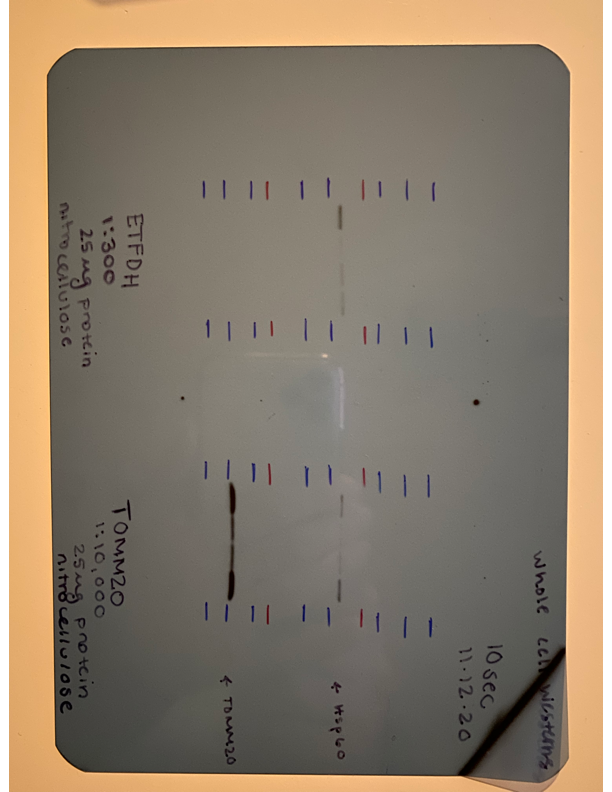


Figure S8: Anti-AK2 antibody (right side)


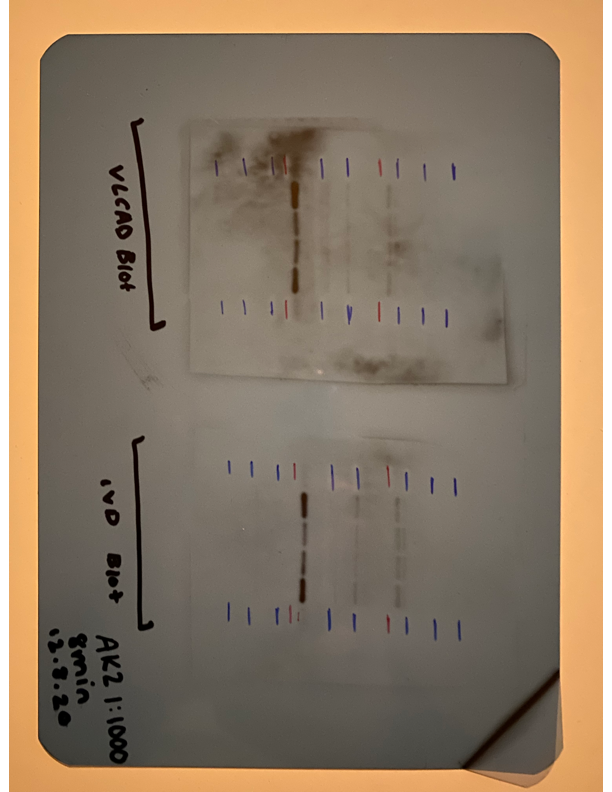


Figure S8: Anti-GAPDH antibody as a loading control (right side)

**
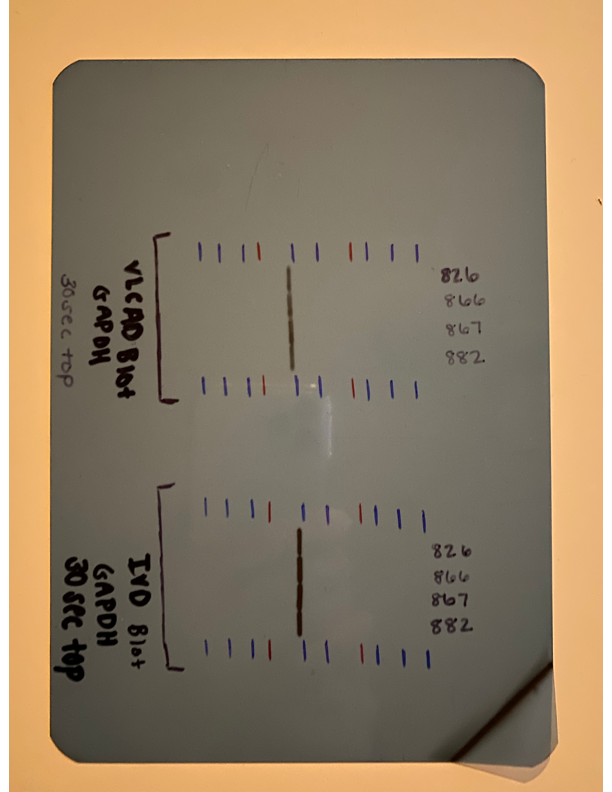
**

**Supplementary Figure S9.** Full uncropped western blots for **Figure 4b**, using an OXPHOS antibody cocktail and anti-MTCO1 antibody on whole cell extracts from fibroblasts from patient and control cell lines. Anti-GAPDH and anti-AK2 antibodies were used as cytosolic and mitochondrial loading controls, respectively. Protein loaded was 25μg.

Figure S9: OXPHOS antibody cocktail and anti-MTCO1 antibody


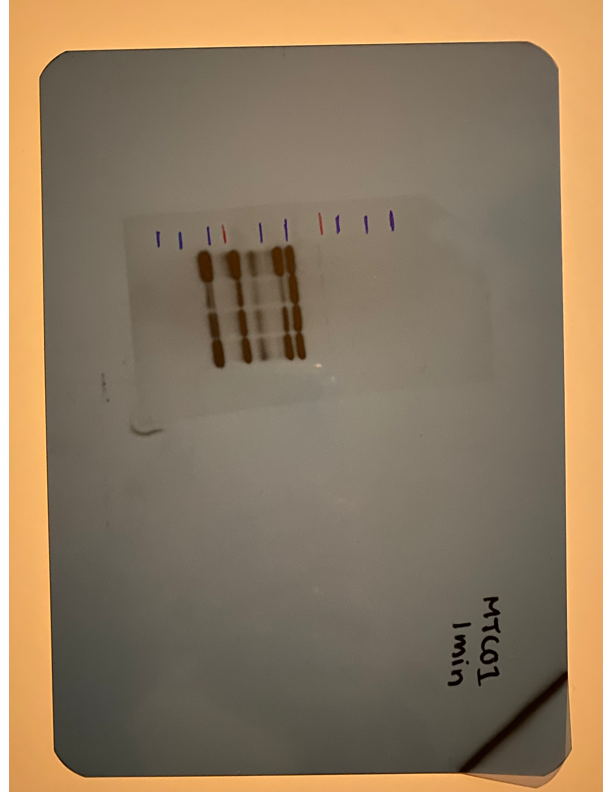


Figure S9: Anti-AK2 antibody


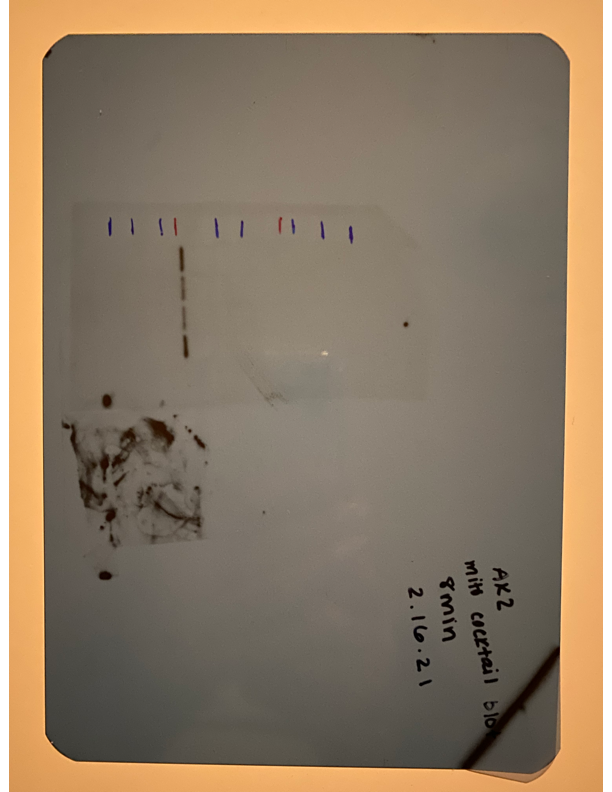


Figure S9: Anti-GAPDH antibody as a loading control. This blot was imaged on the BioRad machine (not X-ray). This is the only image saved for the blot.


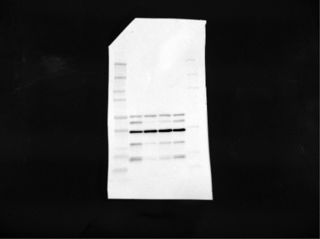

Supplement: Supplementary file 2 — Supplementary Information 2. [file 41598_2022_7076_MOESM2_ESM.docx]
